# A contribution to the anatomy of two rare cetacean species: The hourglass dolphin (*Cephalorhynchus cruciger*) and the spectacled porpoise (*Phocoena dioptrica*)

**DOI:** 10.1002/ar.70045

**Published:** 2025-10-22

**Authors:** Jean‐Marie Graïc, Tommaso Gerussi, Bruno Cozzi, Rebecca M. Boys, Brian Chin Wing Kot, Matthew R. Perrott, Kane Fleury, Tabris Yik To Chung, Henry Chun Lok Tsui, Emma Burns, Trudi Webster, Stuart Hunter, Emma L. Betty, Odette Howarth, Carolina Loch, Sophie White, Steve Dawson, William Rayment, Ros Cole, Derek Cox, Tom Waterhouse, Hannah Hendriks, Anton van Helden, Muriel Johnstone, Ramari Oliphant Stewart, R. Ewan Fordyce, Karen A. Stockin

**Affiliations:** ^1^ Department of Comparative Biomedicine and Food Science University of Padua Padua Italy; ^2^ Cetacean Ecology Research Group, School of Natural Sciences, College of Sciences Massey University Auckland New Zealand; ^3^ Department of Infectious Diseases and Public Health, Jockey Club College of Veterinary Medicine City University of Hong Kong Hong Kong China; ^4^ Department of Chemistry, College of Science City University of Hong Kong Hong Kong China; ^5^ School of Veterinary Science, College of Sciences Massey University Palmerston North New Zealand; ^6^ Tūhura Otago Museum Dunedin New Zealand; ^7^ Department of Conservation Wellington New Zealand; ^8^ Yellow‐Eyed Penguin Trust Dunedin New Zealand; ^9^ Sir John Walsh Research Institute, Faculty of Dentistry University of Otago Otago New Zealand; ^10^ Department of Geology University of Otago Dunedin New Zealand; ^11^ Department of Marine Science University of Otago Dunedin New Zealand; ^12^ Ōraka‐Aparima Rūnaka Aparima (Riverton) New Zealand; ^13^ Te Kauika Tangaroa Charitable Trust Westland New Zealand

**Keywords:** computed‐tomography, histology, morphology, osteology, polar

## Abstract

The anatomical description of the hourglass dolphin (*Cephalorhynchus cruciger*) and the spectacled porpoise (*Phocoena dioptrica*) remains largely unexplored, due to limited specimen availability and preservation challenges. This study employed digital imaging techniques, conventional histology, and computed tomography to provide visualization of anatomical structures for a detailed analysis. We present a comprehensive analysis of the gross macroscopical and microscopical morphology of two hourglass dolphins and four spectacled porpoises. Morphometric measurements and skeletal characteristics aligned with the literature, while internal anatomy (organs and systems) was similar to other odontocetes. Precise and consistent lung measurements were challenging; qualitative assessments indicated relatively large lungs with respect to body size. The spectacled porpoise dorsal fin was uniquely large with a well‐developed blood supply; this is hypothesized to act as a thermoregulatory window, though it may also play a role in sexual display in the case of males. Overall, this study provides new data on the anatomy of the hourglass dolphin and spectacled porpoise, contributing insights that may influence future research on these rare species. The findings highlight the importance of anatomical studies as a basis for explaining evolutionary relationships within cetaceans and their ecological roles in the Southern Ocean ecosystems.

## 1 INTRODUCTION

The hourglass dolphin (*Cephalorhynchus cruciger*, Quoy & Gaimard, 1824), and the spectacled porpoise (*Phocoena dioptrica*, Lahille, 1912) are two species of small (ca. 2 m) cetaceans that inhabit subantarctic and Antarctic waters (Brownell & Donahue, 1999; Cipriano, 2018; Fordyce et al., 1984; Goodall & Brownell Jr., 2018; Hammond et al., 2008). While aspects of their external morphology have been reported (Cipriano, 2018; Goodall & Brownell Jr., 2018; refer to Table 1), internal anatomy has seldom been considered, likely because of their southern, limited geographic range and correspondingly poor access to fresh, intact specimens.

**TABLE 1:** Selected data available on hourglass dolphin and spectacled porpoise specimens available in the published literature.

Parameters	Hourglass dolphin		Spectacled porpoise		References
	Value	*n*	Value	*n*	
Total length (cm)	♂ 162.6–187 ♀ 142–182.9	6 7	♂ 109–224 ♀ 119–203.5 ? 94–101	8 13 11	Goodall et al., 1997; Brownell & Donahue, 1999; Gazitúa et al., 1999; Fernández et al., 2003; Brownell Jr., 1999; Evans et al., 2001; Pinedo et al., 2002
Weight (kg)	♂ 93–100 ♀ 73.5–88	3 2	Mixed sex 1.6 (fetus)—115	7	Goodall et al., 1997; Gazitúa et al., 1999; Fernández et al., 2003; Brownell Jr., 1999; Evans et al., 2001; Pinedo et al., 2002
Condylobasal length (mm)	316–370	11	276–424	54	Goodall et al., 1997; Gazitúa et al., 1999; Cipriano, 2018; Perrin et al., 2000
Visible teeth (per side)	26–34 top 27–35 bottom	6	16–26 top 17–23 bottom		Perrin et al., 2000
Vertebral column	C = 7 Th = 13–14 L = 18–19 Ca = 27+	9	C = 7 Th = 14 L = 14–16 Ca = 32–33		Marchesi et al., 2016; Perrin et al., 2000
Ribs (per side)	12–13	9	13–14		Perrin et al., 2000
Phalangeal formula	I = 2–3 II = 8–11 III = 6–8 IV = 2–4 V = 0–2	6	I = 2 II = 7 III = 4 IV = 3 V = 4		Perrin et al., 2000
Intestine length	18–19.7 m	3			Cipriano, 2018
Number of reniculi	670 (left kidney)	1			Cipriano, 2018
Other particularities	Single vena cava, absence of hepatic sinus	1			Goodall, 1997; Brownell Jr., 1999

**FIGURE 1:**
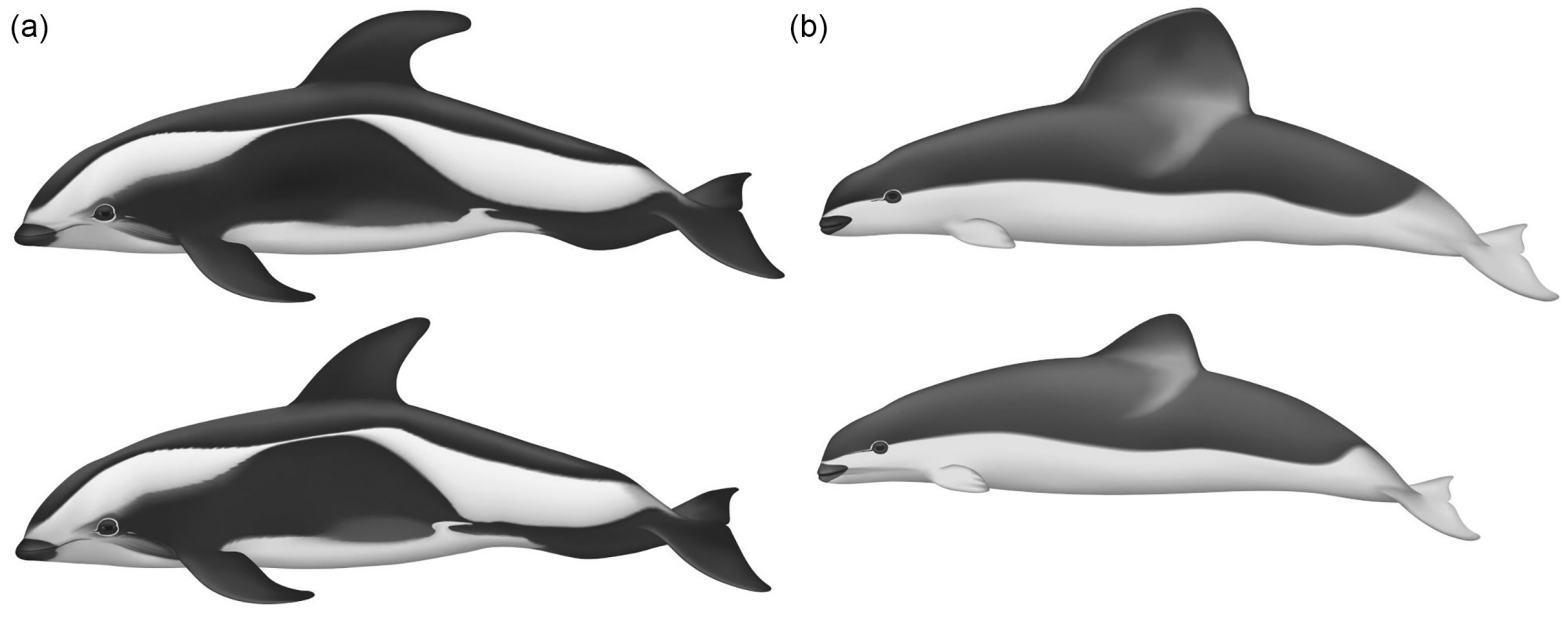
(a) Typical aspect of an adult male (top) and female (bottom) hourglass dolphin (*C. cruciger*) and (b) adult male (top) and female (bottom) spectacled porpoise (*P. dioptrica*). Illustrations courtesy of Uko Gorter (https://ukogorter.com/).

The hourglass dolphin belongs to the family *Delphinidae*, though its taxonomy is still under debate with a recent shift from the genus *Lagenorhynchus to Cephalorhynchus* (Vollmer et al., 2019; see the section “List of Marine Mammal Species and Subspecies” of The Society of Marine Mammalogy for taxonomic update, https://marinemammalscience.org/). Recent genomic analysis offers further insight to the hourglass dolphin and its placement within the *Delphinidae* subfamily (McGrath et al., 2025). The most relevant external characteristics are the distinctive white and black pigmentation of the flanks and the markedly hooked dorsal fin (Figure 1a). The common name arises from the hourglass shaped white marking extending from the beak to the tail flukes (Cipriano, 2018; Jefferson et al., 2015).

The spectacled porpoise belongs to the family *Phocoenidae* and also suffers taxonomic uncertainty as to whether it is best placed within genus *Phocoena* or elsewhere (Jefferson et al., 2015). The species is characterized by a very large dorsal fin with a convex posterior margin; this feature is pronounced in males (Figure 1b). The eye is set within a small oval of black, with a thin dorsal semicircle of white; hence the common name “spectacled” (Goodall & Brownell Jr., 2018; Jefferson et al., 2015).

While the literature describes the external morphology and skeleton of both species (Table 1), information on their visceral anatomy is scarce, possibly due to the limited number of specimens observed and to the decomposition status of the carcasses.

Our anatomical description of these little‐known species yields new data that increase our understanding and may aid future taxonomic resolution. Specifically, our study describes for the first time the gross external morphology and the visceral macro‐ and micro‐anatomy of six specimens (two hourglass dolphin, four spectacled porpoise) examined postmortem.

## 2 MATERIALS AND METHODS

Conventional anatomical methods were applied, including dissection, photography, and histology, alongside post‐mortem computed tomography (PMCT). PMCT examination enhanced three‐dimensional visualization of organs and systems prior to dissection, allowing for volume calculations and comprehensive anatomical insights.

### 2.1 Animal data

We examined six specimens (*n* = 2 hourglass dolphins; *n* = 4 spectacled porpoise) which originated from stranding events in New Zealand between 2010 and 2020 (Table 2).

**TABLE 2:** Specimen and strandings data of hourglass dolphins and spectacled porpoises examined in the study. Decomposition code according to the standards of Ijsseldijk et al., 2019.

Species	Animal ID	Age class	Sex	Stranding date	Location stranding	Dissection date	Decomposition code
Hourglass dolphin	KS10‐28Lc	Adult	M	7 September 2010	Flea Bay (43°86′ S, 173°0′ E), Akaroa, NZ	10 September 2010	2–3
Hourglass dolphin	KS20‐20Lc	Subadult	M	5 August 2020	Orepuki Beach (46°16′ S, 167°43′ E), Te Waewae Bay, NZ	29 September 2020	1–2
Spectacled porpoise	KS14‐45Pd/X2020.76	Adult	M	17 September 2014	Pipikaretu (45°80′ S, 170°7′ E), Otago Peninsula, NZ	19 September 2014	2
Spectacled porpoise	KS14‐37Pd/X2020.77	Subadult	M	2 October 2014	Caroline Bay Beach (44°38′ S, 171°24′ E), Timaru, NZ	2 October 2014	2
Spectacled porpoise	KS15‐29Pd/VT3347	Juvenile	F	9 August 2015	Bayleys Beach, (43°49′ S, 172°36′ E) Kaitorete Spit, Canterbury, NZ	19 August 2015	3
Spectacled porpoise	KS20‐07Pd	Adult	M	14 January 2020	Washdyke Lagoon, (44°21' S 171°15' E) Canterbury, NZ	22 January 2020	2

### 2.2 PMCT scan data

PMCT scanning of the first hourglass dolphin (KS10‐28Lc) was performed with a LightSpeed VCT CT scanner (GE Healthcare, USA), using the exposure parameters: 120 kV, 100 mA, 0.63 mm slice thickness, and sFOV of 45 cm. PMCT of the second hourglass (KS20‐20Lc) was conducted using a CT 5000 Ingenuity CT scanner (Philips, Netherlands), with exposure parameters: 120 kV, 20 mA, 0.90 mm slice thickness, and sFOV of 50 cm. Given the width of the pectoral fins, both forelimbs were removed and scanned independently. Three of the spectacled porpoises underwent PMCT scanning: KS14‐37Pd/X2020.77 and KS14‐45Pd/X2020.76 were scanned using a LightSpeed Pro 16 (GE Medical Systems); exposure parameters for KS14‐45Pd/X2020.76 were 120 kV, 390 mA, and 1.25 mm slice thickness, while for KS14‐37Pd/X2020.77 parameters were: 120 kV, 270 mA, and 1.25 mm slice thickness. Due to logistical difficulties, the dorsal fin of KS14‐45Pd/X2020.76 was scanned separately in the LightSpeed Pro 16 with scan parameters of 120 kV, 270 mA, and slice thickness of 1.25 mm; this allowed for specific imaging of the dorsal fin structure. PMCT scanning of KS20‐07Pd was conducted using an Optima CT660 (GE Medical Systems), with exposure parameters: 120 kV, 480 mA, and 0.63 mm slice thickness. Due to logistical difficulties during cadaver transportation, the tail stock was removed and scanned separately in KS20‐07Pd (exposure parameters remained the same), rendering it difficult to determine the precise number of lumbar vertebrae caudal to the dorsal fin. CT data from KS20‐07Pd and KS20‐20Lc cases was assessed using PMCT methodology (Granados‐Zapata et al., 2022; Kot et al., 2020) and were used to guide the necropsy, thus improving findings of the conventional necropsy. Both scans were viewed using the TeraRecon iNtuition workstation (TeraRecon, San Mateo, CA, USA). Morphological and volumetric examinations were performed using Slicer 3D (https://www.slicer.org/) by both automatic thresholding, using presets such as bone or air, and manual segmentation of the organs.

### 2.3 Dissection

Dissections occurred at the Cetacean Pathology Unit, Massey University Auckland (KS10‐28Lc, KS20‐20Lc, KS20‐07Pd), AgResearch Invermay Campus, Dunedin (KS14‐37Pd/X2020.77) and Tūhura Otago Museum, Dunedin (KS14‐45Pd/X2020.76, KS15‐29Pd/VT3347), New Zealand, following standardized sampling techniques (Geraci & Lounsbury, 2005; IJsseldijk et al., 2019). A more restricted examination was performed on the first hourglass dolphin (KS10‐28Lc, no ingoa [cultural] name assigned) in order to preserve the integrity of the cadaver for cultural display. However, a full systematic dissection for KS20‐20Lc (ingoa name, “Harua‐tai‐nui”) and all spectacled porpoises was permitted. Standardized histological tissue samples of key organs were fixed in 10% buffered formalin solution, then trimmed, paraffin embedded, and subsequently sectioned (8 μm) on a rotary microtome. Sections were stained with conventional hematoxylin–eosin stain, or with Masson's trichrome.

The pectoral fins were analyzed morphometrically, following Benke (1993). In addition, we measured the ratio between the flipper maximum length and total body length by dividing the “flipper length external” and “total length” measurements of each animal individually and then calculating the mean and standard deviation among species. The condylo‐basal length was also measured following Mead and Potter (1995).

## 3 RESULTS

### 3.1 External appearance

Weight and morphometric measurements of each specimen are reported below in Table 3.

**TABLE 3:** Weight (kg) and morphometrics (cm) of examined specimens.

	Hourglass dolphin (*Cephalorhynchus cruciger*)		Spectacled porpoise (*Phocoena dioptrica*)			
Parameter	KS10‐28Lc	KS20‐20Lc	KS14‐45Pd/X2020.76	KS14‐37Pd/X2020.77	KS15‐29Pd/VT3347	KS20‐07Pd
Weight (kg)	78	n/a	120	96	26	n/a
Total length (cm)	170.5	183	215	200	125	210
Snout‐anus	121.5	134	149	144	88	155
Snout‐genital slit	102.5	113	126	117	81	137.5
Snout‐navel	78	82.5	n/a	n/a^c^	60	95.5
Snout‐origin flipper	32	35.5	36	30	22	29.5
Snout‐origin dorsal fin	67	83^a^	83 (anterior insertion of fin)	78	51	83
Snout‐tip dorsal fin	104.5	119	128	n/a	70	119.5
Snout‐blowhole	19	22		n/a	14	18
Snout‐corner mouth	18.5	20.5	12	7.8	7	9
Genital slit	13	15	11	26	9	11.5
Rostrum length	2	n/a	n/a	n/a	15	n/a
Corner mouth‐eye	5	5.5	20 (snout)	7.8	8.5	11
Eye‐blowhole	20.5	18.5	n/a	12	8	17.5
Dorsal fin height	16	22	27	28.5	10	26
Dorsal fin flat base	30.5	42	49	50	25	49
Fluke width	47	53.5	53	46	32	49
Fluke length	17	20	17	18	14	17
Fluke notch	2	2.5	n/a	3	2.5	3
Flipper length internal	33.5	26	n/a	16	11	11
Flipper length external	18	35	23	21.6	17	24
Flipper length width	11	13	10.5	9.6	7	10
Girth eye	73	76	n/a	64	47	72
Girth flippers (back)	98	112.5	100^a^	94	65	105
Girth navel	105.5	124	116	n/a^b^	73	n/a
Girth anus	59.5	69	81	70	43	83
Blubber (mid dorsal) dorsal	0.9	1.8	2	2.1	1.4	1.8
Blubber (mid dorsal) lateral	1.4	1.4	2.36	2.14	1.4	1.57
Blubber (mid dorsal) ventral	1.4	1.8	2.36	2	1.5	1.9

^a^ Anterior insertion of the fin.

^b^ With shark bite 108 based on measurements from right side.

^c^ Navel is mid dorsal fin, cannot accurately measure this on mature adult male spectacled porpoise.

**FIGURE 2:**
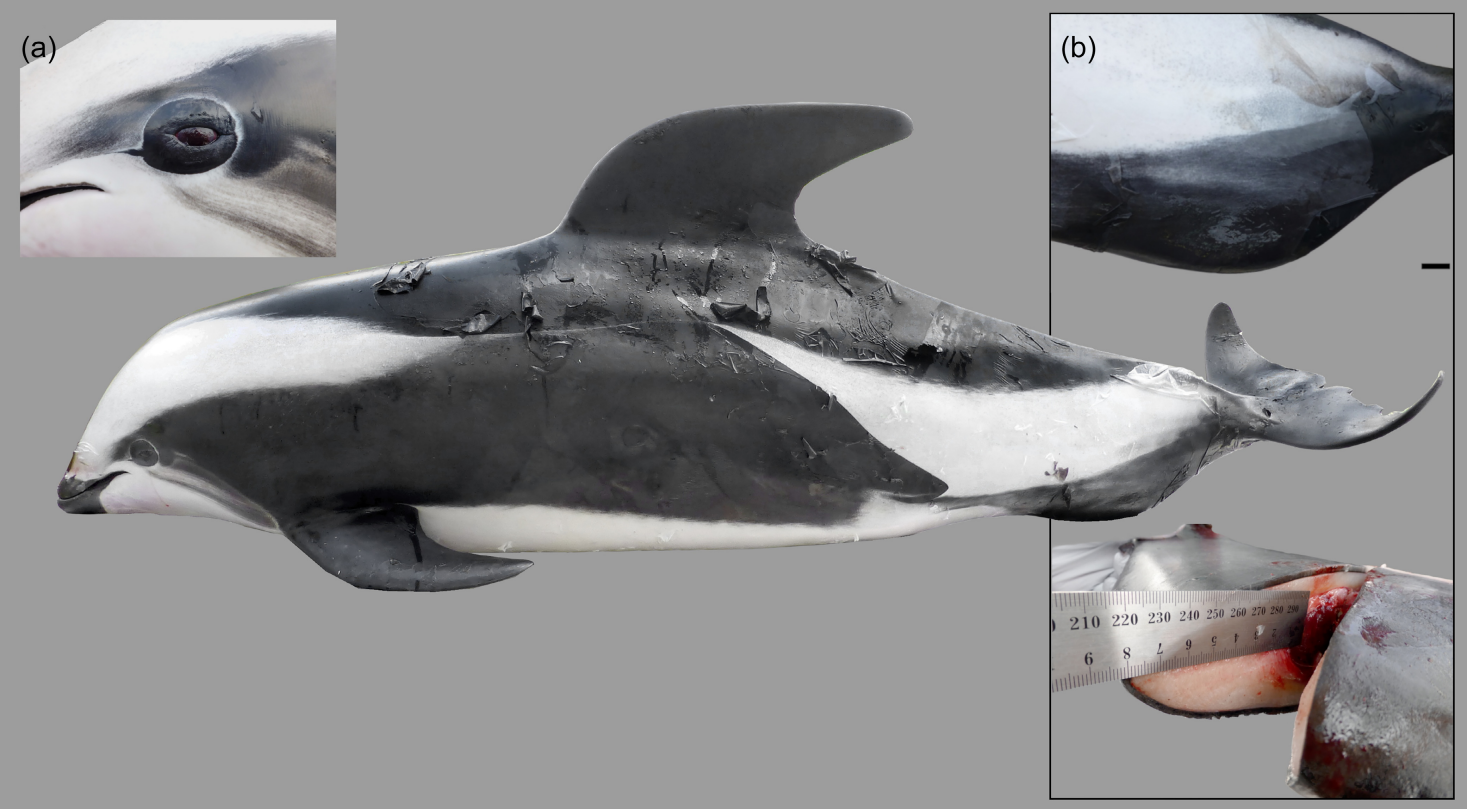
Adult male specimen of hourglass dolphin (*C. cruciger*) KS10‐28Lc. Pigmentation pattern with the characteristic shape of an hourglass. (a) White line around the eye spot and (b) Caudal keel showing presence of connective tissue and absence of any musculature.

The hourglass dolphins had a smoothly rounded head and no protruding rostrum. The dorsal fin had a long base, was falcate, and notably tapered caudally, ending with a bluntly pointed tip. The pectoral fins displayed a narrower base and a notch on the caudal border adjoining the body. The caudal fin, or fluke, was relatively wide and thin. The caudal peduncle had an obvious ventral keel (Figure 2b), composed of dense connective tissue and blubber, with no additional musculature (Figure 2b). The characteristic color pattern was similar to that described in the literature (see Figures 1 and 2). In common with the spectacled porpoise, the hourglass dolphins had an incomplete white line dividing the black eye spot from the black pattern of the head (Figure 2a).

**FIGURE 3:**
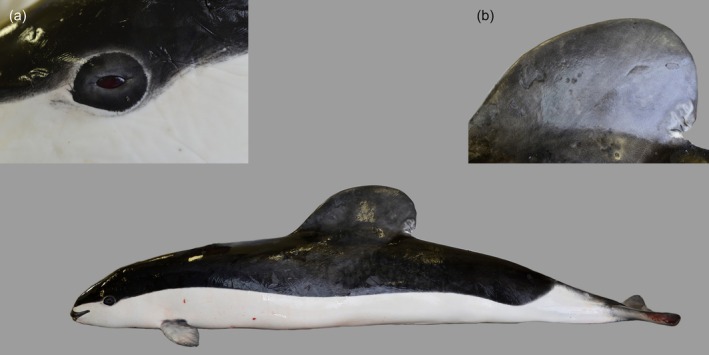
Adult male spectacled porpoise (*P. dioptrica*) KS14‐45Pd/X2020.76. (a) Distinctive white rim around the black eye patch and (b) the unique large dorsal fin with its typical profile in males.

The spectacled porpoises showed a typical porpoise‐like body form, having a squat and tapered body, a clear division between dorsal black and ventral white, rounded pectoral fins, and a distinctively large dorsal fin with a convex trailing edge. The white line around the eye spot was incomplete (Figure 3).

The hourglass dolphin appeared to be wider transversely and shorter in its axial dimension than the spectacled porpoise, which was narrower transversely and longer in its axial dimension.

In both hourglass dolphins, we detected four small pits in the skin of the rostrum (ca. 1 cm apart), likely representing remnants of the vibrissae. These were not observed in any of the examined spectacled porpoises.

### 3.2 Osteology

**FIGURE 4:**
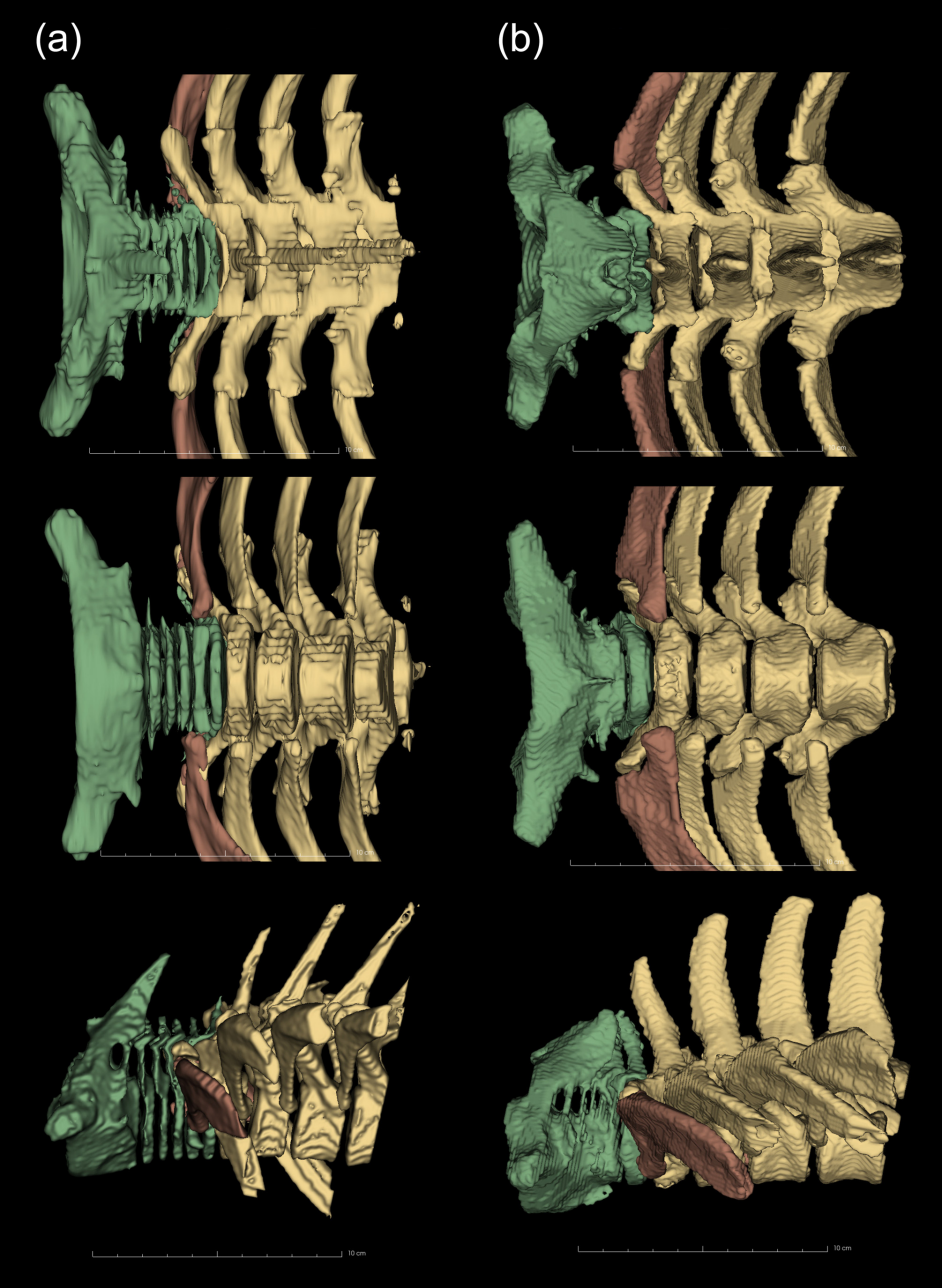
3D rendering of the cervical and thoracic vertebrae with corresponding attached ribs of the (a) adult hourglass dolphin (*C. cruciger*) KS20‐20Lc and (b) spectacled porpoise (*P. dioptrica*) KS14‐45Pd/X2020.76. The cervical vertebrae were segmented in green (C1‐2, C3, C4, C5, C6, and C7); in yellow the first four thoracic vertebrae and ribs. In red, the first rib. Top: dorsal view; middle: ventral view; bottom: left lateral view.

In the hourglass dolphin, the first two cervical vertebrae (C1 and C2) were fused, while in the spectacled porpoise, C1 to C6 were fused (Figure 4). In both species, the atlas was large and relatively flat, while the remaining six vertebrae were tightly aligned with their bodies joined together. For vertebral formulae and details, see Table 4.

**TABLE 4:** Number of vertebrae per section, number of ribs, and phalangeal formula (first and last number represent left and right flipper, respectively) by specimen.

	Hourglass dolphin (*Cephalorhynchus cruciger*)		Spectacled porpoise (*Phocoena dioptrica*)			
	KS10‐28Lc	KS20‐20Lc	KS14‐45Pd/X2020.76	KS14‐37Pd/X2020.77	KS15‐29Pd/VT3347	KS20‐07Pd
CB length	319.6	362.8	307.2	293.4	239	289.6
Cervical	7	7	7	n/a	7	7
Thoracic	13	13	13	n/a	13	13
Lumbar	15	19	n/a	n/a	17	n.d.
Coccygeal	nd	16 + 20	32	n/a	37	27
Ribs	13	13	13	n/a	n/a	15?
Phalangeal formula	n/a	I = 3; 2 II = 10; 10 III = 7; 7 IV = 3; 3 V = 3; 3	I = 1; 1 II = 6 (R) III = 5; 4 IV = 3; 3 V = 1; 1	n/a	(R only) I = 1 II = 6 III = 5 IV = 4 V = 1	(R only) I = 2 II = 6 III = 5 IV = 4 V = 1

Abbreviation: CB, condylo‐basal.

**FIGURE 5:**
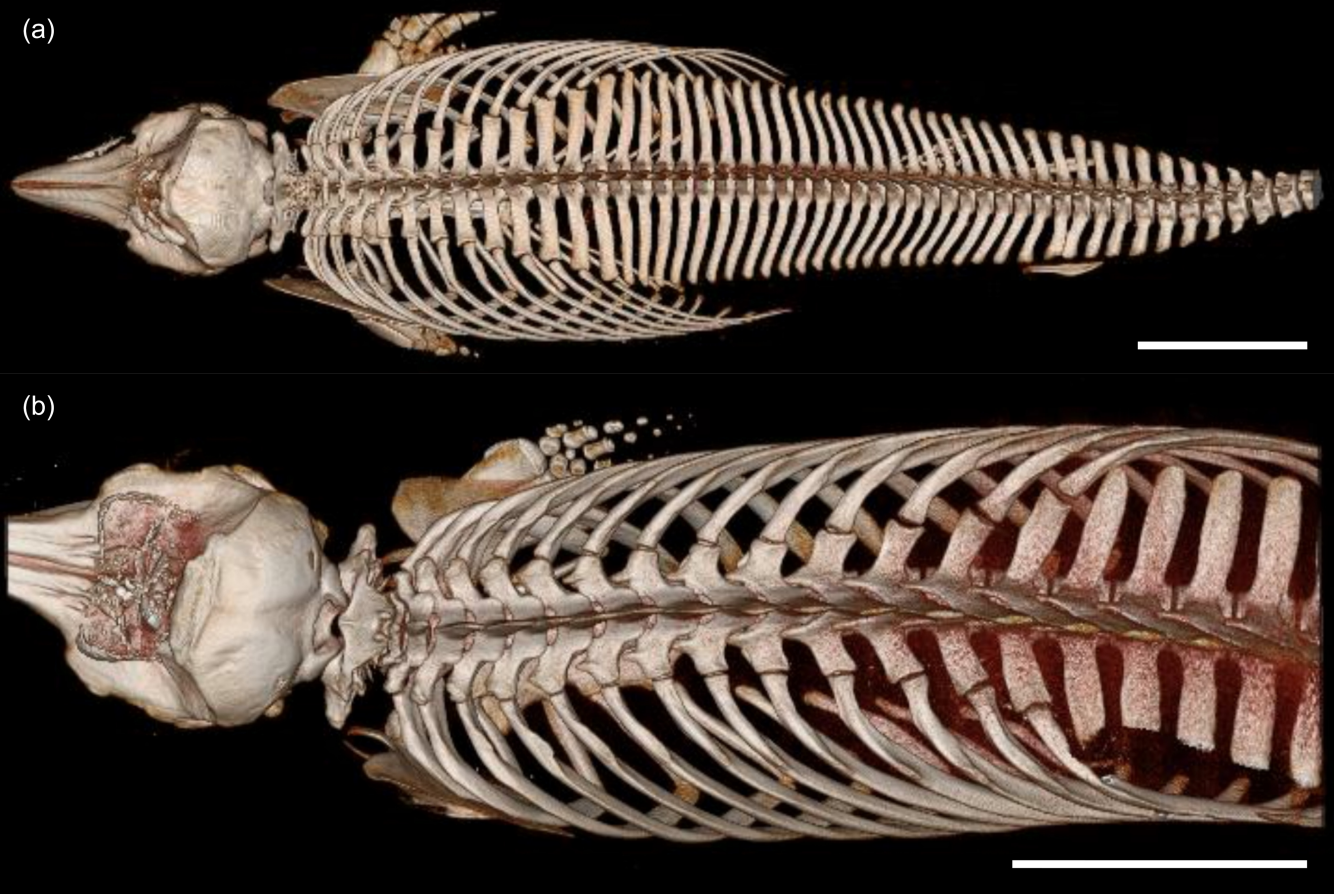
Representation of the skeleton up to the (a) caudal vertebrae of hourglass dolphin (*C. cruciger*) KS20‐20Lc and (b) lumbar vertebrae of spectacled porpoise (*P. dioptrica*) KS20‐07Pd, as displayed by 3D rendering of the CT scans. In (B) there are also hyperintense areas at the level of the nasal sacs, most likely due to the presence of sand. In addition, the left lumbar transverse processes appear truncated, which we confirm is a scanning artifact. Scale bar = 20 cm.

Both hourglass dolphin specimens had 13 thoracic vertebrae (Th) and a corresponding number of ribs. Ribs 1 to 5 articulated with the sternum. The distal extremities of ribs 6 to 8 joined the distal bony aspect of the ribs rostral to them, while ribs 9 to 13 did not connect to the sternum. Ribs 1 to 6 articulated directly with the *foveas* of the transverse processes of the corresponding vertebrae (unlike in other mammals, in which they articulate with the *foveas* of two consecutive vertebrae, Cozzi et al., 2017). Interestingly, we observed the head of the first rib to articulate cranially to the 6th and 7th cervical vertebrae. In the spectacled porpoises, ribs 1 to 4 were connected to the sternum, ribs 5 to 8 were connected to the ribs rostral to them, and the remaining ones were free of any relationship to the sternum (Figure 5).

**FIGURE 6:**
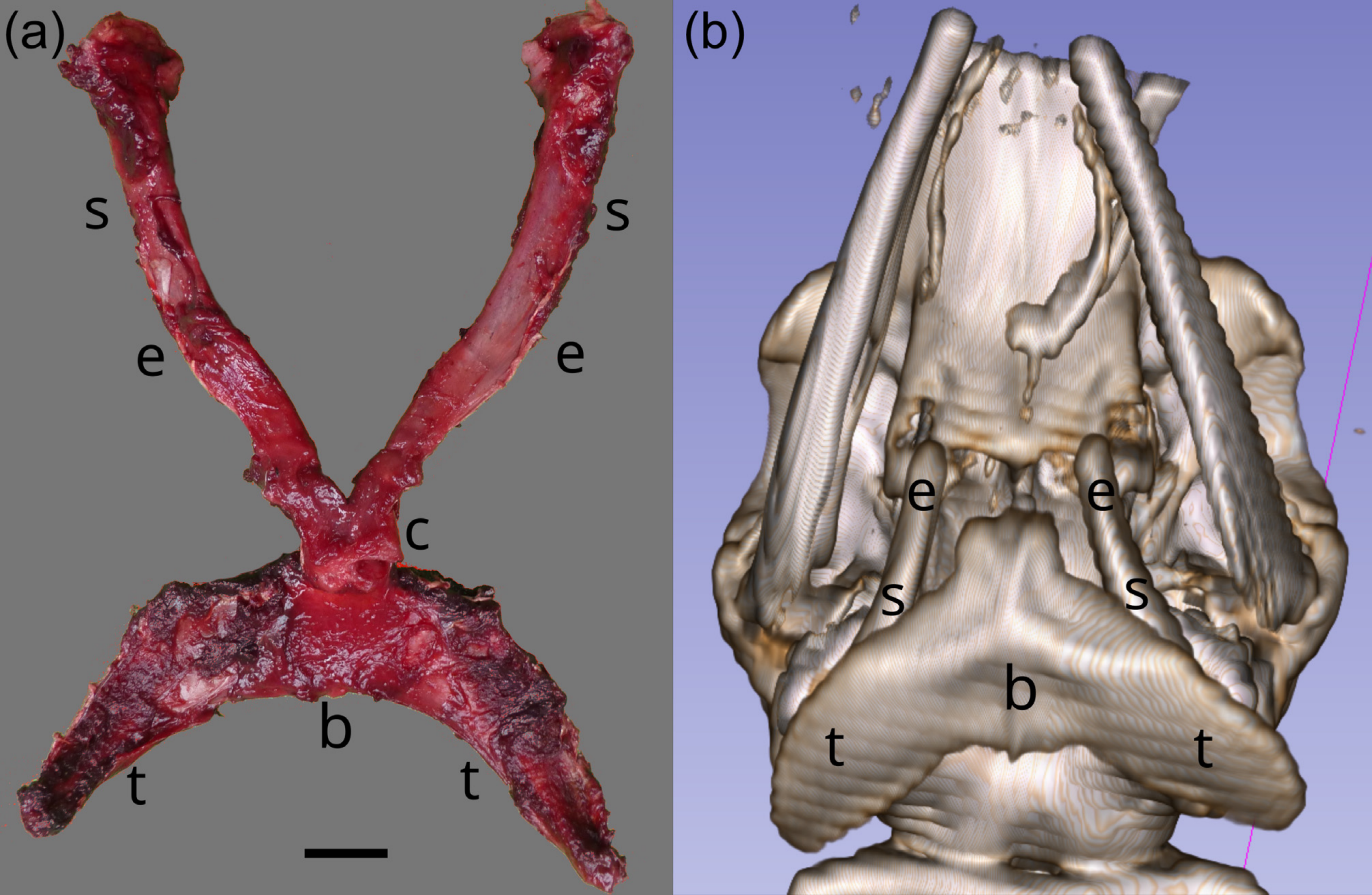
(a) Isolation of the hyoid apparatus in a dorsal view and extension of the stylohyal bones rostrally of hourglass dolphin (*C. cruciger*) KS20‐20Lc and (b) 3D reconstruction of the skeleton in a ventral view including the hyoid bone of spectacled porpoise (*P. dioptrica*) KS20‐07Pd. B, basihyal; C, ceratohyal; E, epihyal; sh, stylohyal; T, thyrohyal. Scale bar = 2 cm.

The hyoid complex in hourglass dolphin KS20‐20Lc displayed a typical *Cephalorhynchus* (previously named as *Lagenorhynchus*) basihyal connected to the stylohyals, as described by Yablokov et al. (1974). In the spectacled porpoises, the basihyal and thyrohyal were fused in a porpoise‐like unique flat bone, articulated cranially with the two stylohyals that extended caudally (Figure 6).

**FIGURE 7:**
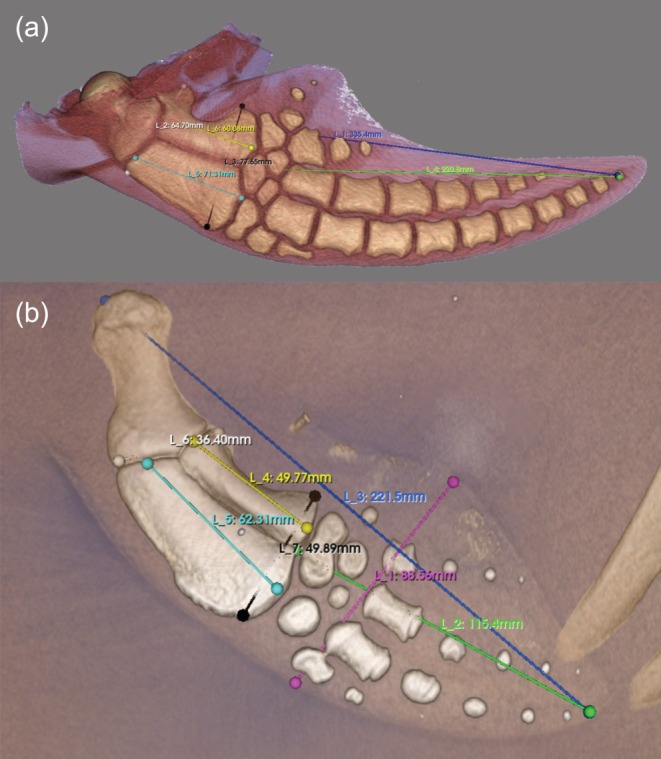
Visualization of the skeletal characteristics with principal measurements of 3D reconstructions of (a) left pectoral flipper in lateral view of hourglass dolphin (*C. cruciger*) KS20‐20Lc and (b) left pectoral flipper in lateral view of the spectacled porpoise (*P. dioptrica*) KS20‐07Pd.

The pectoral fins of the hourglass dolphin were long and slender, with flat bones and numerous phalanges. Conversely, the spectacled porpoise flippers were more tapered in shape, with the smaller and fewer phalanges providing a rounded shape toward the tip. The ratio of flipper length to body length in the hourglass dolphins (*n* = 2) was 14.8% (SD = 6.1%), while the ratio in all spectacled porpoises (*n* = 4) was on average 11.63% (SD = 1.35%). When excluding the juvenile, the mean ratio only among adults (*n* = 3) was 10.98% (SD = 0.40%). Pectoral morphometry is detailed in Figure 7 and Table 5.

**TABLE 5:** Morphometry of the pectoral fins.

Measurement (cm)	Hourglass dolphin (*Cephalorhynchus cruciger*)		Reference (*Tursiops truncatus*) (Benke, 1993)	Spectacled porpoise (*Phocoena dioptrica*)	
	KS20‐20Lc			KS20‐07Pd	KS15‐29Pd/VT3347
Side (right and left)	R	L	R	R	R
FLB (flipper width)	111	111	77	88.56	70.6
FLL (flipper length)	342	335	n/a	221.5	165
ML (manus length)	233	229	70	115.4	84.6
RL (radius length)	73.1	70.8	75	62.31	48.4
RUD (radius and ulna distal width)	80.3	77.6	70	49.89	42.8
RUP (radius and ulna proximal width)	63.4	64.2	64	36.4	38.6
UL (ulna length)	65.7	64.8	66	49.77	40.2

### 3.3 Tympano‐periotic complex

**FIGURE 8:**
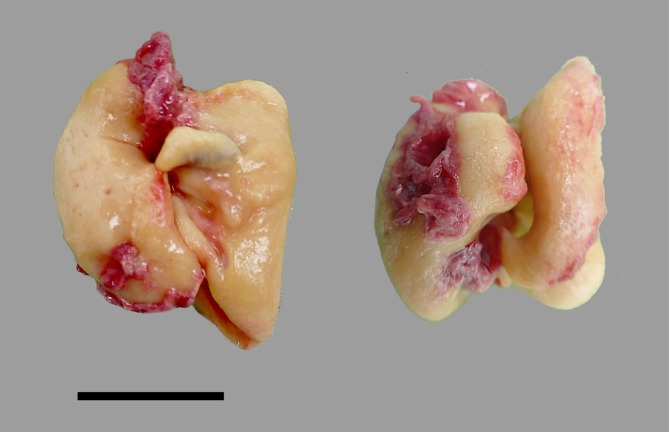
Left tympano‐periotic complex of hourglass dolphin (*C. cruciger*) KS20‐20Lc in its lateral (left) and medial (right) view. Scale bar = 1 cm.

In hourglass dolphin KS20‐20Lc, the right and left tympano‐periotic complex weighed 18 and 17 g, respectively (Figure 8). For a full reporting of measurements, refer to Table 6.

**TABLE 6:** Morphometry of the tympanic‐periotic complex.

	Hourglass dolphin (*Cephalorhynchus cruciger*)		Spectacled porpoise (*Phocoena dioptrica*)		
Parameter	KS10‐28Lc	KS20‐20Lc	KS14‐45Pd/X2020.76	KS14‐37Pd/X2020.77	KS20‐07Pd
T R measurements (mm)	35 × 21	35 × 19	32 × 15	32 × 18	30 × 18
T L measurements (mm)	34 × 18	34 × 19	31 × 12	31 × 15	31 × 19
P R measurements (mm)	30 × 22	29 × 15 × 8	36 × 19	31 × 15	32 × 18
P L measurements (mm)	31 × 20	29 × 18 × 12	36 × 20	32 × 20	31 × 20

*Note*: Measurements are shown as length × width × depth.

Abbreviations: L, left; P, periotic bone; R, right; T, tympanic bone; TPC, tympanic‐periotic complex.

### 3.4 Visceral anatomy

#### 3.4.1 Respiratory system

**FIGURE 9:**
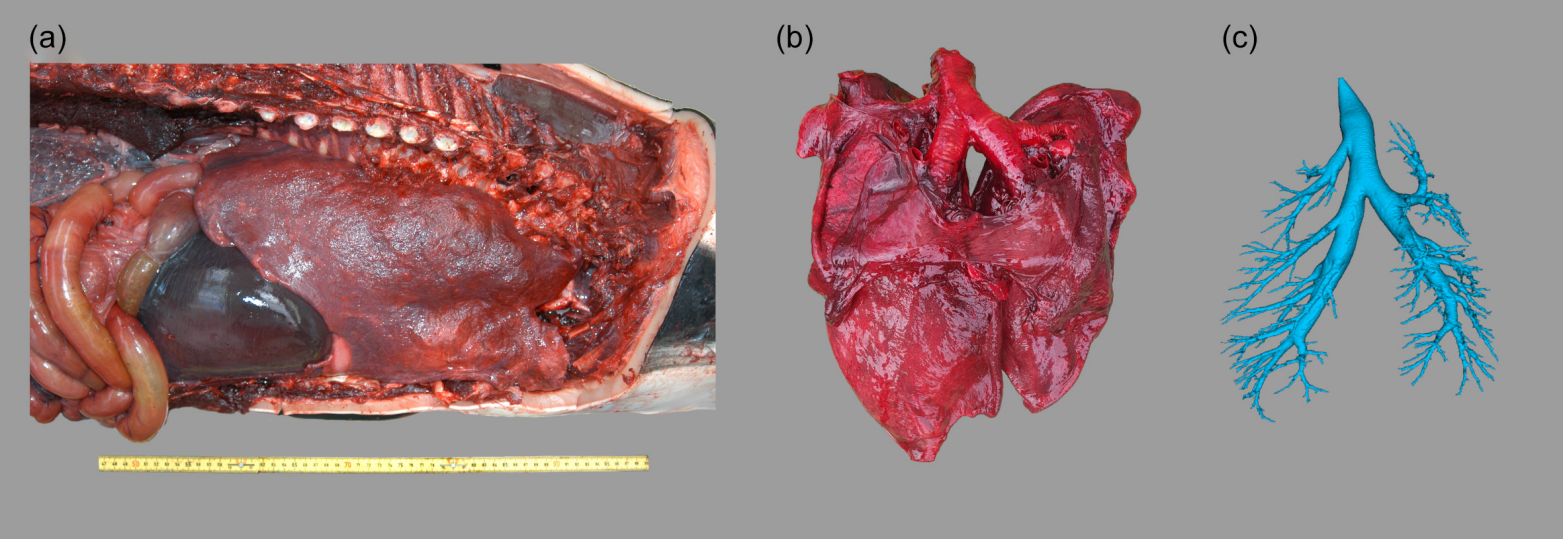
Thorax with lungs in their topographical location without ribs (a), isolated ventral aspect (b), and as a 3D reconstruction, showing the trachea and bronchial tree in ventral view in hourglass dolphin (*C. cruciger*) KS10‐28Lc. Scale shown is common to all images.

**FIGURE 10:**
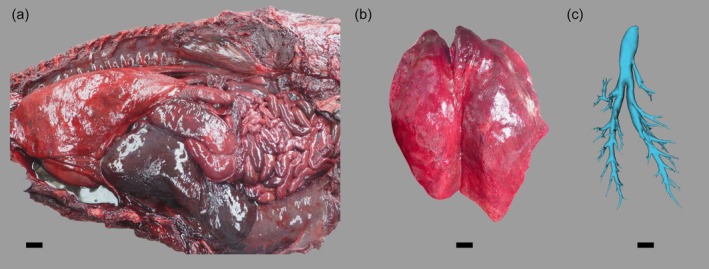
Representation of spectacled porpoise (*P. dioptrica*) KS20‐07Pd lungs (a) in their topographical location and (b) after removal in dorsal view. (c) 3D reconstruction of the trachea and bronchial tree in dorsal view in spectacled porpoise KS20‐07Pd. Scale bar = 2 cm.

**FIGURE 11:**
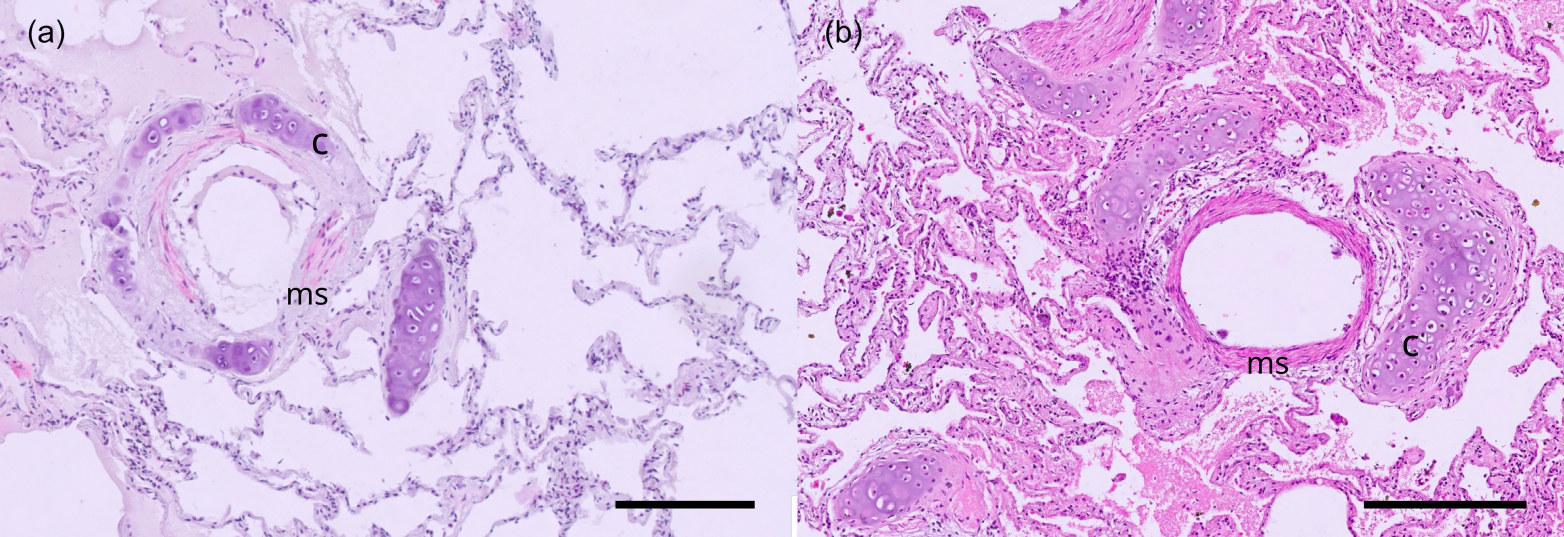
Photomicrograph of the (a) hourglass dolphin (*C. cruciger*) KS20‐20Lc and (b) spectacled porpoise (*P. dioptrica*) KS20‐07Pd, small bronchioles showing the presumed myoelastic sphincters (ms) surrounded by cartilage (C). Scale bar = 100 μm. Hematoxylin–eosin stain.

In both species, we found robust tracheal cartilaginous rings, visible bronchial cartilages down to the smallest distinguishable airway, a right tracheal bronchus slightly cranial to the primary bronchial division, and no apparent lobation of either the left or right lung. Some parameters can be found in Table 7. In hourglass dolphins, both the lungs terminated in alignment with rib 12. In spectacled porpoises, the left lung terminated approximately at rib 12, with the right lung aligned with rib 10 (Figures 9 and 10). Histologically, in both species, it was possible to observe presumed myoelastic sphincters surrounded by cartilage in the smaller bronchioles (Figure 11).

**TABLE 7:** Lung parameters. Measurements are shown as (craniocaudal) length × (dorsoventral) height × (lateromedial) width.

Parameter	Hourglass dolphin (*Cephalorhynchus cruciger*)	Spectacled porpoise (*Phocoena dioptrica*)		
	KS20‐20Lc	KS14‐37Pd/X2020.77	KS15‐29Pd/VT3347	KS14‐45Pd/X2020.76
Total weight (g)	3100	600.3 (L); 673 (R)	328.2 (L); 365.35 (R)	1456 (L); 1078 (R)
Total volume (L)	3.4	n/a	n/a	n/a
Right lung measurements (mm)	440 × 190 × 98	400 × 120	n/a	n/a
Left lung measurements (mm)	420 × 170 × 75	400 × 130	n/a	n/a

#### 3.4.2 Circulatory and lymphatic systems

##### Heart and vessels

In both species, the heart was relatively flat dorsoventrally and triangular in shape, with wide and flat auricles. The left ventricle was larger, with a thicker wall compared to the right. Table 8 summarizes the most important measurements in some specimens.

**TABLE 8:** Heart parameters. Measurements shown as width × length × diameter.

Parameter	Hourglass dolphin (*Cephalorhynchus cruciger*)	Spectacled porpoise (*Phocoena dioptrica*)		
	KS20‐20Lc	KS14‐37Pd/X2020.77	KS15‐29Pd/VT3347	KS14‐45Pd/X2020.76
Weight (g)	296.6	562.9	269.6	1037 (approx. vol. of 1857 cm^3^)
Measurements (mm)	n/a	180 × 220	n/a	192 × 210 × 45.6

**FIGURE 12:**
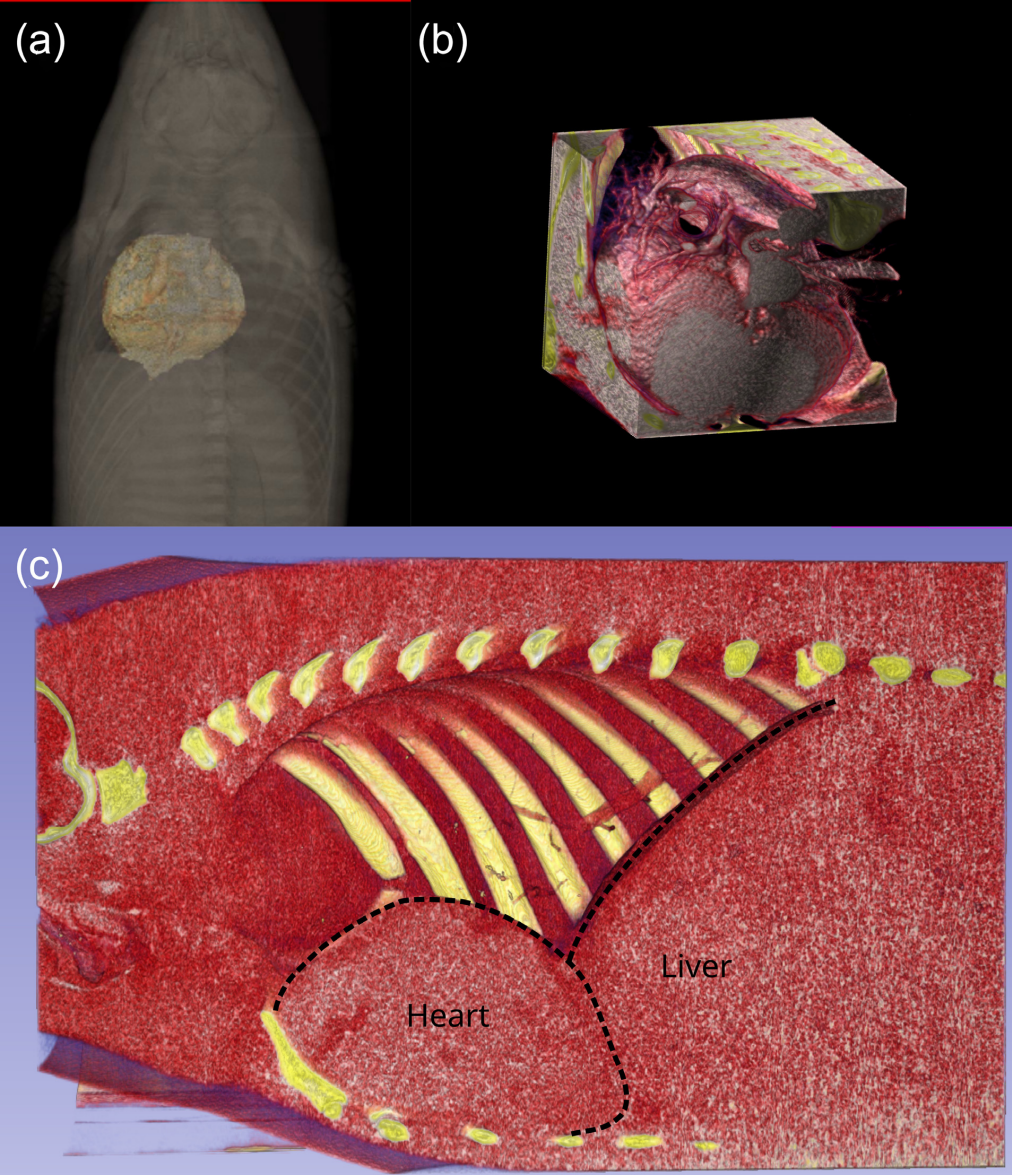
3D reconstruction of the heart in (a, b) hourglass dolphin (*C. cruciger*) KS20‐20Lc and (c) spectacled porpoise (*P. dioptrica*) KS20‐07Pd. (a) Dorsal view of the heart, showing its topography in the thoracic cavity; (b) illustrates the heart in isolation, with the aortic arch and onset of aorta shown; and (c) presents a 3D model of the heart, sectioned along the sagittal plane, revealing detailed views of the right side of the heart and the liver.

The heart of both hourglass dolphins was located between the intercostal spaces 1–5, lying on the sternum with its major axis oriented laterally, so that the right and left ventricles aligned to the right sides, respectively (Figure 12a,b). The paraconal groove was relatively deep with large arteries covered in fat. The diameter of the aorta was approximately 3 cm.

The hearts of spectacled porpoises KS20‐07Pd and KS14‐45Pd/X2020.76 were located on the sternum between intercostal space 1 and 4 (Figure 12c), and had an approximate volume of 1320 cm^3^. The heart of KS20‐07Pd weighed 813 g, with an aorta diameter of 3.9 cm recorded. Several vertebral arteries extended from the aorta to the thoracic rete mirabile along its course in the thorax.

**FIGURE 13:**
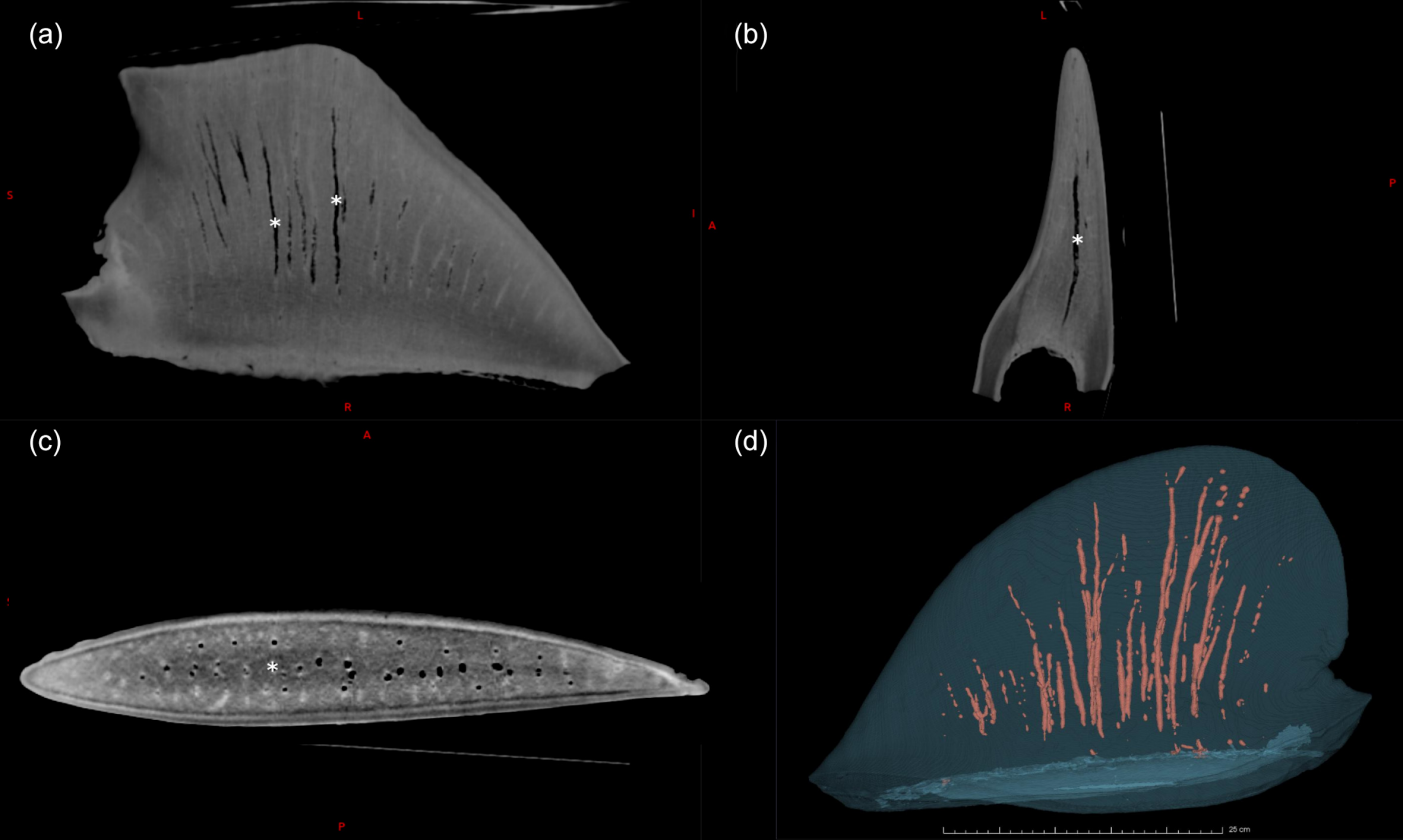
CT scan of the dorsal fin of spectacled porpoise (*P. dioptrica*) KS14‐45Pd/X2020.76. (a), (b) and (c) show the sagittal, transversal, and axial planes at its base, respectively. (d) The 3D reconstruction with the render of the arteries. In (a), (b) and (c), the asterisks highlight the central arteries. These are shown as hypodense (dark) regions. The circumferential veins surrounding the arteries, due to their weaker wall and size, may have collapsed, thus appearing as small hyperdense areas. Conversely, the peripheral veins were visible as hyperdense areas.

In spectacled porpoise KS14‐45Pd/X2020.76, it was also possible to remove the dorsal fin and scan it independently (Figure 13). The images displayed the characteristic pattern of the countercurrent exchange system in cetacean appendages, featuring central arteries located along the midline of the dorsal fin and branching dorsally, encircled by circumferential veins (Figure 13). We hypothesized that the central arteries originating from the body were represented by the hypodense areas in the CT scan, as these structures possessed rigid walls and may have lost their blood content, resulting in air‐filled cavities while maintaining their structural integrity. However, instead of observing a singular line of arteries, we identified multiple lines, exhibiting an inhomogeneous distribution (Figure 13c). The 3D rendering revealed large vessels only in the central section of the dorsal fin, with scarcity in the rostral and caudal areas. Bifurcation varied, with some vessels bifurcating at the base of the dorsal fin while other vessels bifurcated more toward the tip, and others at mid‐height (Figure 13d). Cranial and caudal arteries tended to curve cranially and caudally at the tip of the fin, respectively, while central arteries ran perpendicular to the fin. Conversely, it was not possible to observe any similar pattern of vasculature in the dorsal fin of the hourglass dolphins, which appeared as a single and homogeneous gray area.

#### 3.4.3 Spleen

**FIGURE 14:**
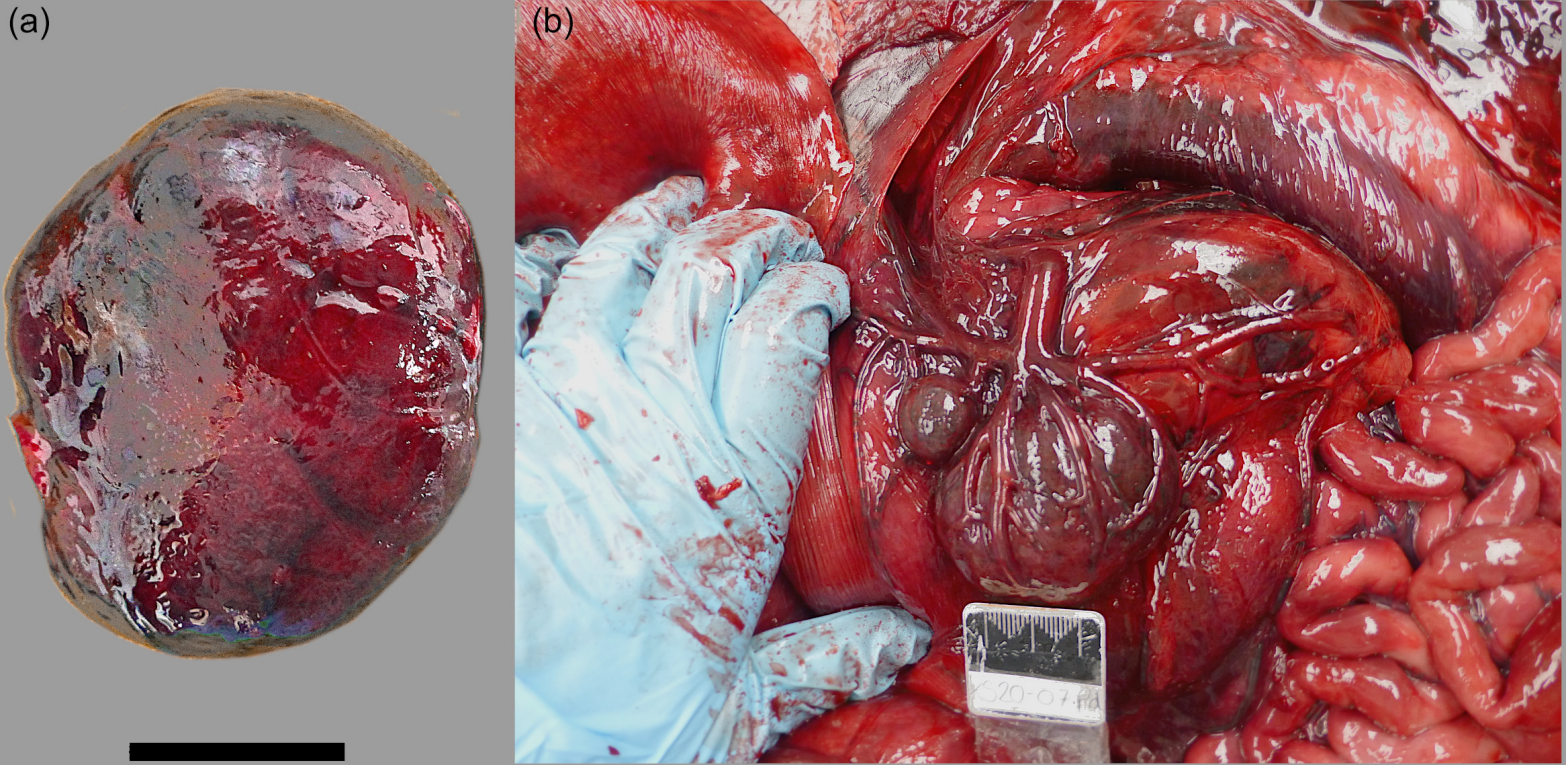
Spleen of porpoise (*P. dioptrica*) KS20‐07Pd (a), with the presence of an accessory spleen (b) shown to the left of the primary spleen. Scale bar = 2 cm.

**FIGURE 15:**
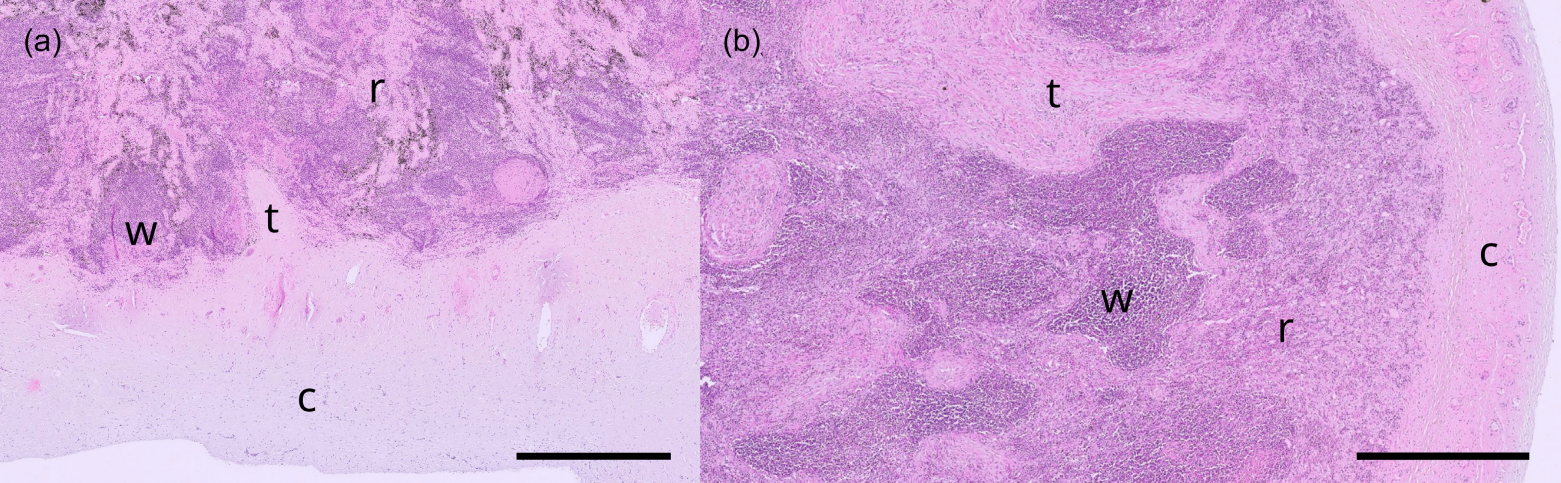
Photomicrograph of the (a) hourglass dolphin (*C. cruciger*) KS20‐20Lc and (b) spectacled porpoise (*P. dioptrica*) KS20‐07Pd spleen. Note the capsule (C) sending trabecule (T) into the parenchyma, in turn divided into white pulp (W) and red pulp (R). Scale bar = 1000 μm. Hematoxylin–eosin stain.

In both specimens, the spleen was observed, with primary parameters provided in Table 9. In spectacled porpoise KS20‐07Pd, an accessory spleen was further recorded (Figure 14). In hourglass dolphin KS20‐20Lc, prescapular lymph node weights and measurements were 15 g (L) and 20 g (R), and 51 × 40 × 11 mm (L) and 72 × 39 × 15 mm (R), respectively. Histologically, in both species, the spleen was composed of an external capsule, which also sent trabeculae into the underlying parenchyma, a white pulp composed of immune cells distributed in the red pulp (Figure 15). Mesenteric lymph nodes were present at the root of the mesentery.

**TABLE 9:** Spleen parameters. Measurements shown as length × width × diameter (mm).

	Hourglass dolphin (*Cephalorhynchus cruciger*)	Spectacled porpoise (*Phocoena dioptrica*)		
	KS20‐20Lc	KS15‐29Pd/VT3347	KS14‐45Pd/X2020.76	KS20‐07Pd
Weight (g)	20	19.93	16	27
Measurements (mm)	63 × 40 × 15	40 × 35	n/a	59 × 48 × 17

### 3.5 Digestive system

#### 3.5.1 Mouth and upper digestive tract

While most teeth were intact in the hourglass dolphin KS10‐28Lc, most teeth in the hourglass dolphin KS20‐20Lc were worn to the gumline (LR: 1–11; LL: 1–13; UR: 1; UL: 1–5 and 9–24). This indicates an older specimen, which was further supported in the mineralization of the pectoral limb (Figure 7) in KS20‐20Lc. Teeth of KS15‐29Pd were only partially erupted, and teeth of KS14‐37Pd/X2020.77 were not examined. The dental formula of each examined specimen can be viewed in Table 10.

**TABLE 10:** Dental formula of selected specimens examined.

Parameter	Hourglass dolphin (*Cephalorhynchus cruciger*)		Spectacled porpoise (*Phocoena dioptrica*)		
	KS10‐28Lc	KS20‐20Lc	KS20‐07Pd	KS14‐45Pd/X2020.76	KS15‐29Pd/VT3347
Teeth UR	24	19	18	21	18
Teeth LR	27	26	19	19	19
Teeth UL	25	24	19	23	18
Teeth LL	25	27	18	17	17

Abbreviation: LL, lower left; LR, lower right; UL, upper left; UR, upper right.

**FIGURE 16:**
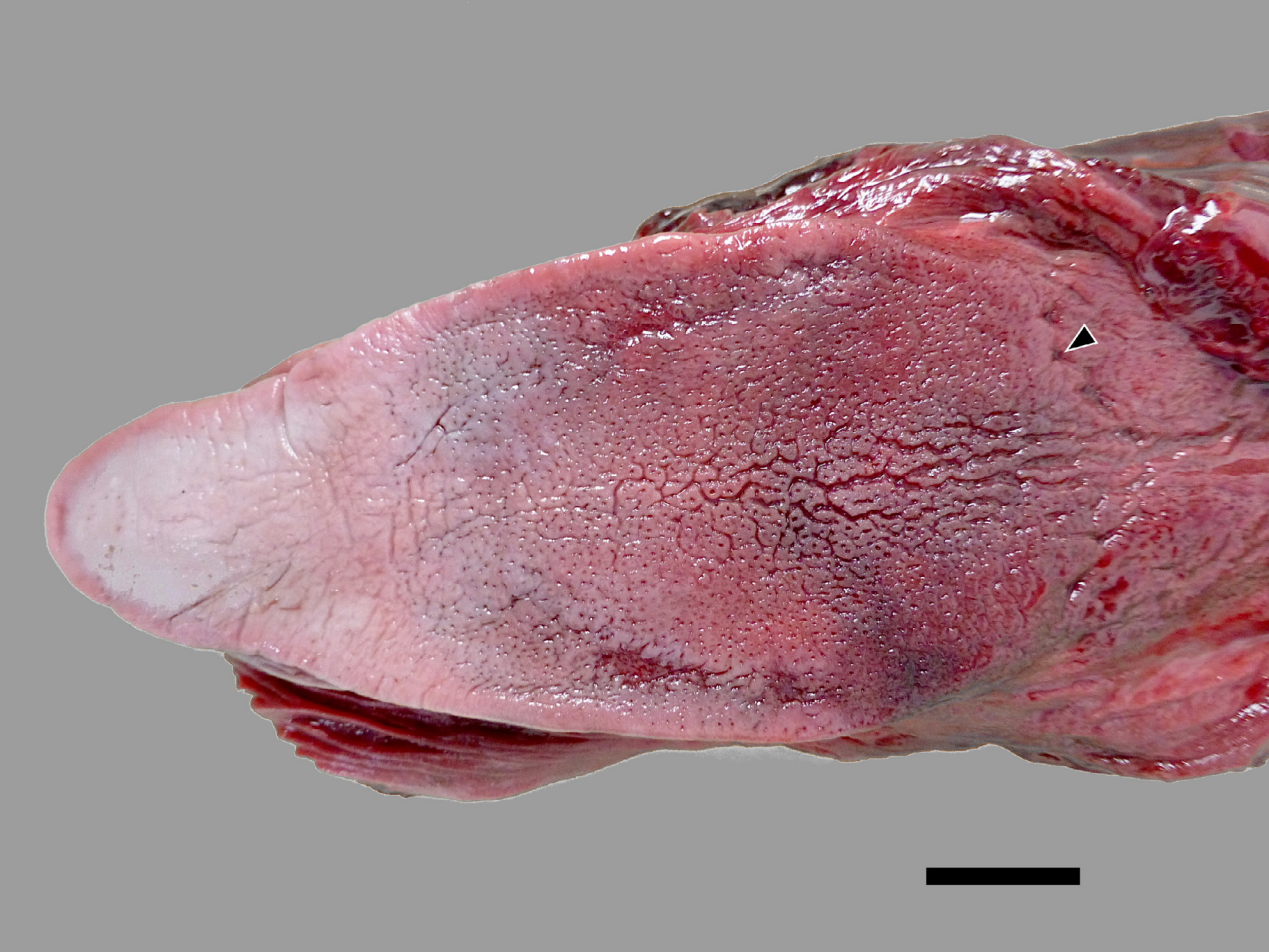
Tongue of the hourglass dolphin (*C. cruciger*) KS20‐20Lc. Arrowhead indicates vallate papilla in a V oriented toward the pharynx. Scale bar = 2 cm.

The pointed tongue in KS20‐20Lc measured 13.3 (L) × 6.3 (W) cm, with no anterolateral papillae. Six vallate papillae were present at the root of the tongue, arranged in a V with the vertex oriented toward the pharynx (arrowhead in Figure 16).

#### 3.5.2 Stomach complex and intestine

**FIGURE 17:**
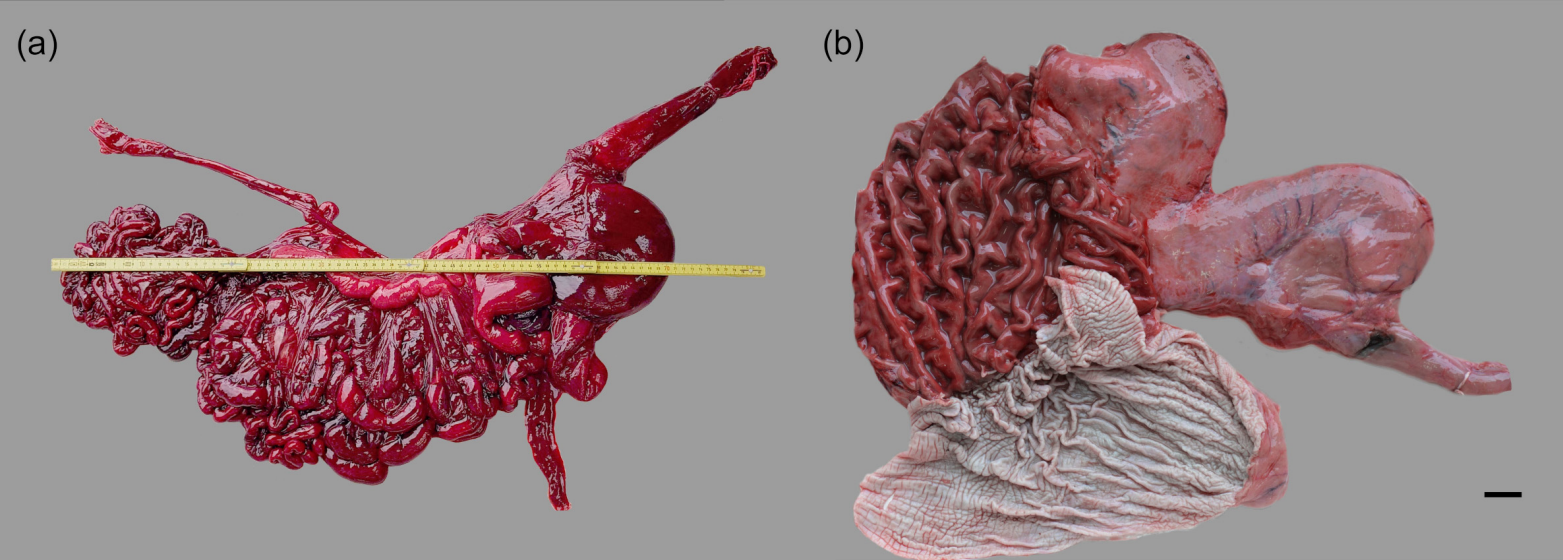
Gastrointestinal components of the digestive system. (a) Whole gastrointestinal system of hourglass dolphin (*C. cruciger*) KS20‐20Lc, starting from the esophagus and terminating at the rectum. (b) Gastric chambers of the spectacled porpoise (*P. dioptrica*) KS14‐45Pd/X2020.76. E, esophagus; F, forestomach; I, intestine; ln, mesenteric lymph nodes; M, main stomach; P, pyloric stomach. Scale bar = 1 cm.

**FIGURE 18:**
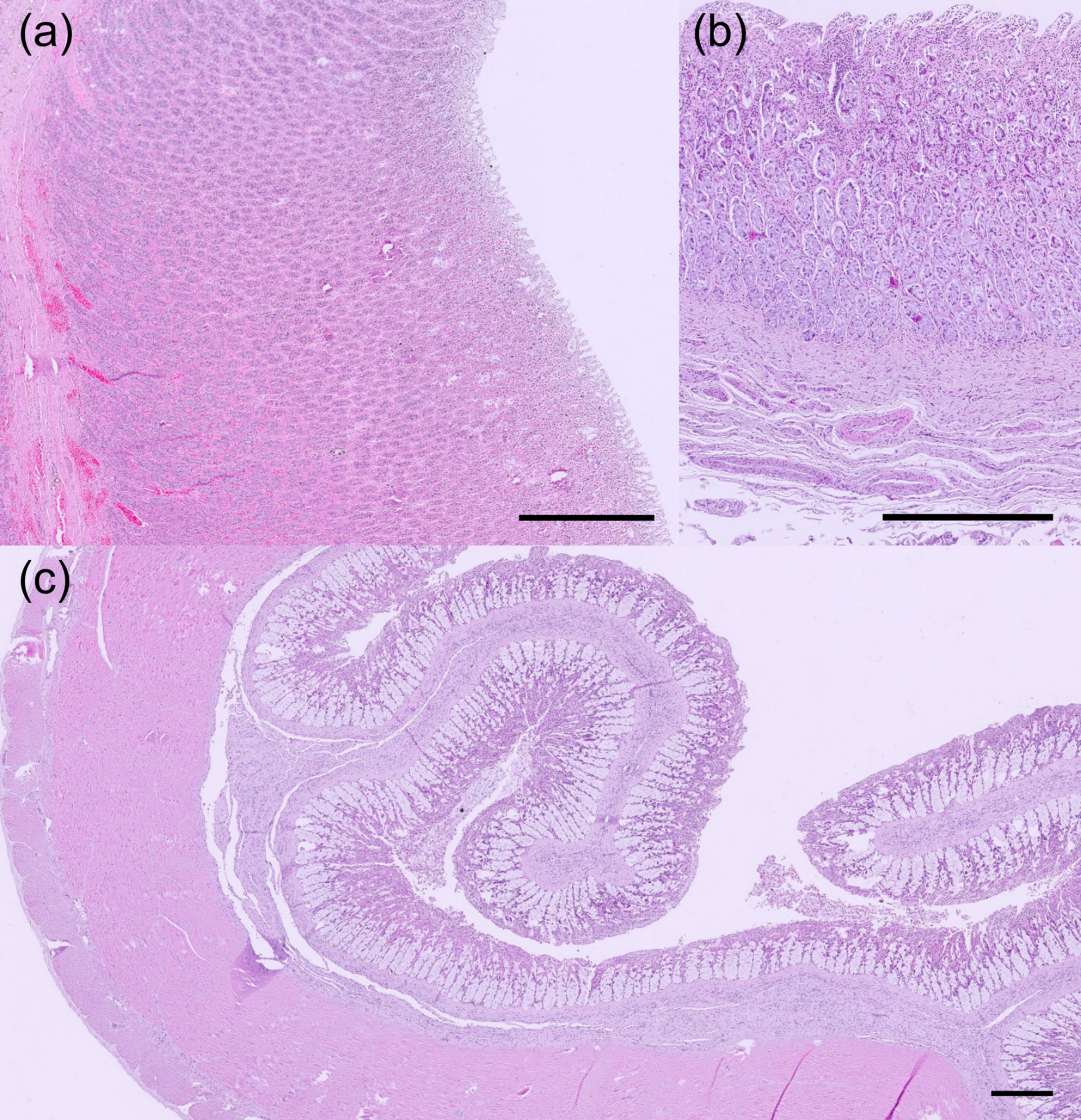
Microphotograph of the different mucosae of (a) spectacled porpoise (*P. dioptrica*) KS20‐07Pd main stomach; (b) hourglass dolphin (*C. cruciger*) KS20‐20Lc pyloric stomach and (c) spectacled porpoise (*P. dioptrica*) KS20‐07Pd jejunum. Scale bar = 200 μm. Hematoxylin–eosin stain.

The stomach chambers of all specimens resembled the typical delphinid pattern: one forestomach, one main stomach, and one pyloric stomach. Similarly, in both species, the intestine was a unique “tube” without macroscopical distinction between small and large parts, and lacking a caecum. The mesenteric lymph nodes were clearly identified (Figure 17). Microscopically, the main stomach (second chamber) mucosa was thick with the typical pattern of other mammals, such as the presence of glandular cells secreting mucus and HCl (Figure 18a); the pyloric stomach (third chamber) mucosa was thinner with columnar epithelium and tubular glands (Figure 18b). Finally, the mucosa of the jejunum was characterized by the typical presence of villi (Figure 18c).

#### 3.5.3 Liver

**FIGURE 19:**
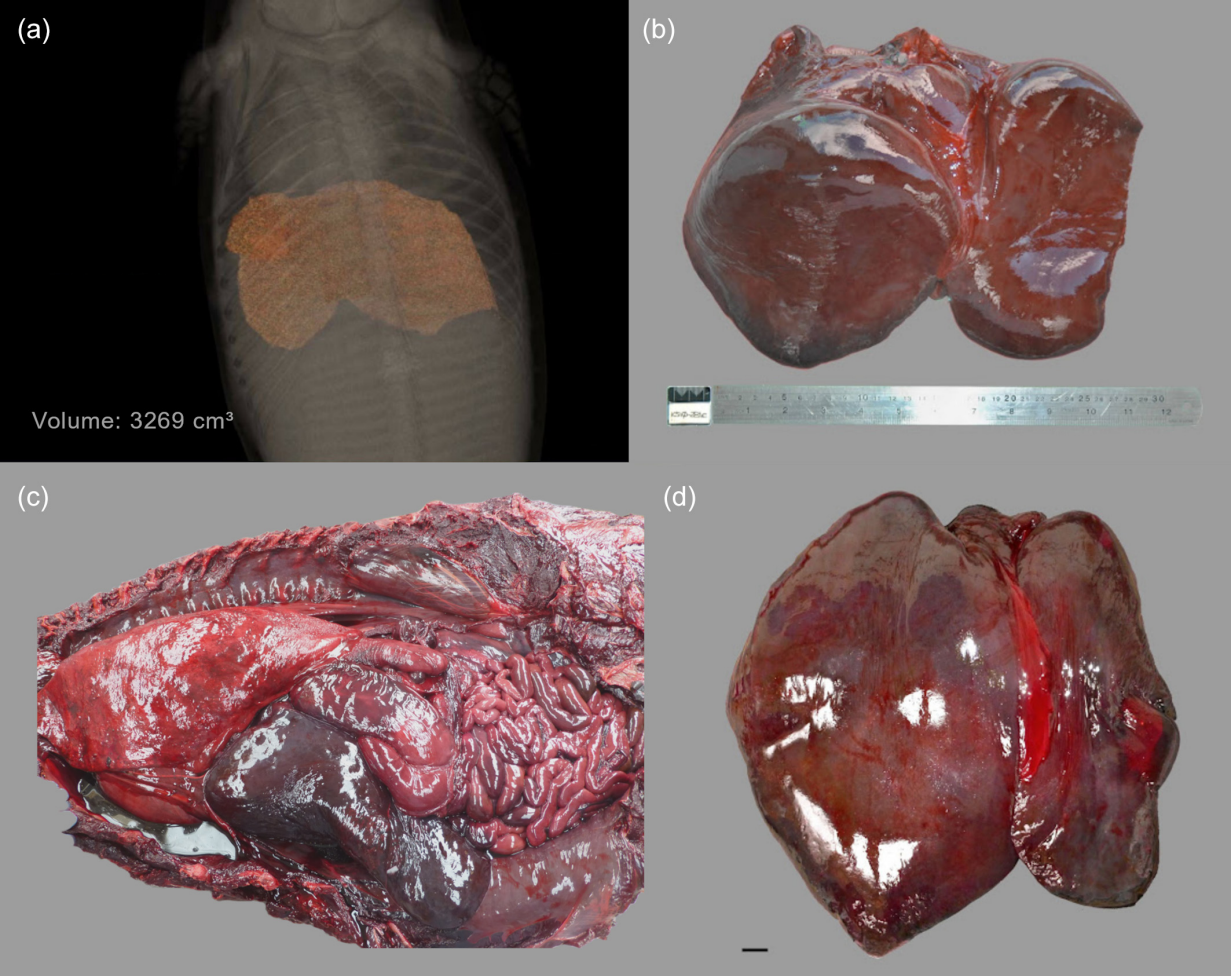
Representation of the liver in the (a, b) hourglass dolphin (*C. cruciger*) KS20‐20Lc and (c, d) spectacled porpoise (*P. dioptrica*) KS20‐07Pd showed their diaphragmatic faces, with the right lobe on the left and the left lobe on the right. (a) and (c) showed the liver in its topographical location within the abdominal cavity while (b) and (d) showed the extracted and isolated organ. Note the difference between the large right lobe compared to the smaller left lobe. Scale bar of (D) = 1 cm.

In the two hourglass dolphins, liver position ran from the 10th thoracic to the 2nd lumbar vertebrae, on the ventral half of the abdominal cavity (Figure 19a). In KS10‐28Lc, the right lobe extended considerably further than the left, which is consistent with its topography and the position (leftward) of the stomachs. The diaphragmatic surface of the right lobe was expanded cranially and contained most of the mass of the organ. The right lobe was separated from the left by a thin falciform ligament, which terminated in a sheet covering the cranial portion of the stomachs (Figure 19b). This division by the ligament was less evident in the KS20‐20Lc, where the liver appeared almost divided in even halves along the longitudinal plane, although the right half was thicker in depth than its left counterpart.

In the spectacled porpoises, the liver was positioned approximately between thoracic rib 8 and lumbar vertebra 4. A highly developed right lobe, compared to the more inferior left lobe, was noted, without the aforementioned diaphragmatic expansion observed in the two hourglass dolphin specimens. The falciform ligament still clearly divided the main left and right lobes of the liver (Figure 19c,d). In both species, we also could not find any venous sinus, apparent lobulation, nor gallbladder.

**FIGURE 20:**
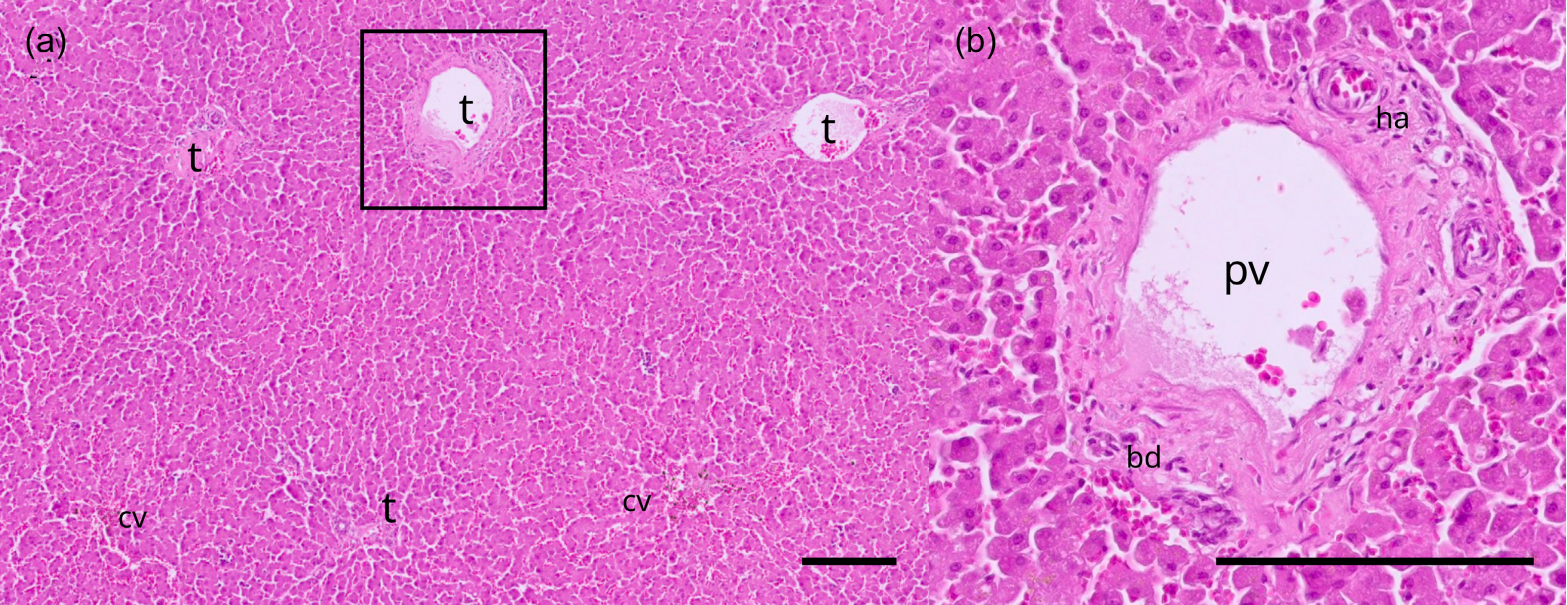
Microphotograph of the hourglass dolphin (*C. cruciger*) KS28‐10Lc liver. (a) low‐magnification image showing the overall hepatic organization in central veins (cv) surrounded by portal triads (T). (b) high‐magnification image of a portal triad of (a) showing the portal vein (pv), hepatic artery (ha) and bile duct (bd). Scale bar = 200 μm. Hematoxylin–eosin stain.

Histologically, in the hourglass dolphin, the hepatic parenchyma was formed of lobules, not easily distinguishable due to the absence of connective septa and clear central veins. However, portal triads, in turn composed of a portal vein, hepatic artery, and bile duct, were identified (Figure 20). No definite muscular wall surrounding the portal vein was found.

Details on the dimensions and weight of the liver for three specimens (KS10‐28Lc, KS20‐20Lc, and KS20‐07Pd) are reported in Table 11.

**TABLE 11:** Liver morphometry in hourglass dolphin and spectacled porpoise. Measurements shown as width × length × height.

	Hourglass dolphin (*Cephalorhynchus cruciger*)		Spectacled porpoise (*Phocoena dioptrica*)			
Parameter	KS10‐28Lc	KS20‐20Lc	KS20‐07Pd	KS14‐45Pd/X2020.76	KS14‐37Pd/X2020.77	KS15‐29Pd/VT3347
Weight (g)	1865	1964	3855	4143	2368	861
Measurements (mm)	n/a	272 × 309 × 42	n/a	n/a	n/a	n/a

### 3.6 Endocrine system

#### 3.6.1 Adrenal glands

**FIGURE 21:**
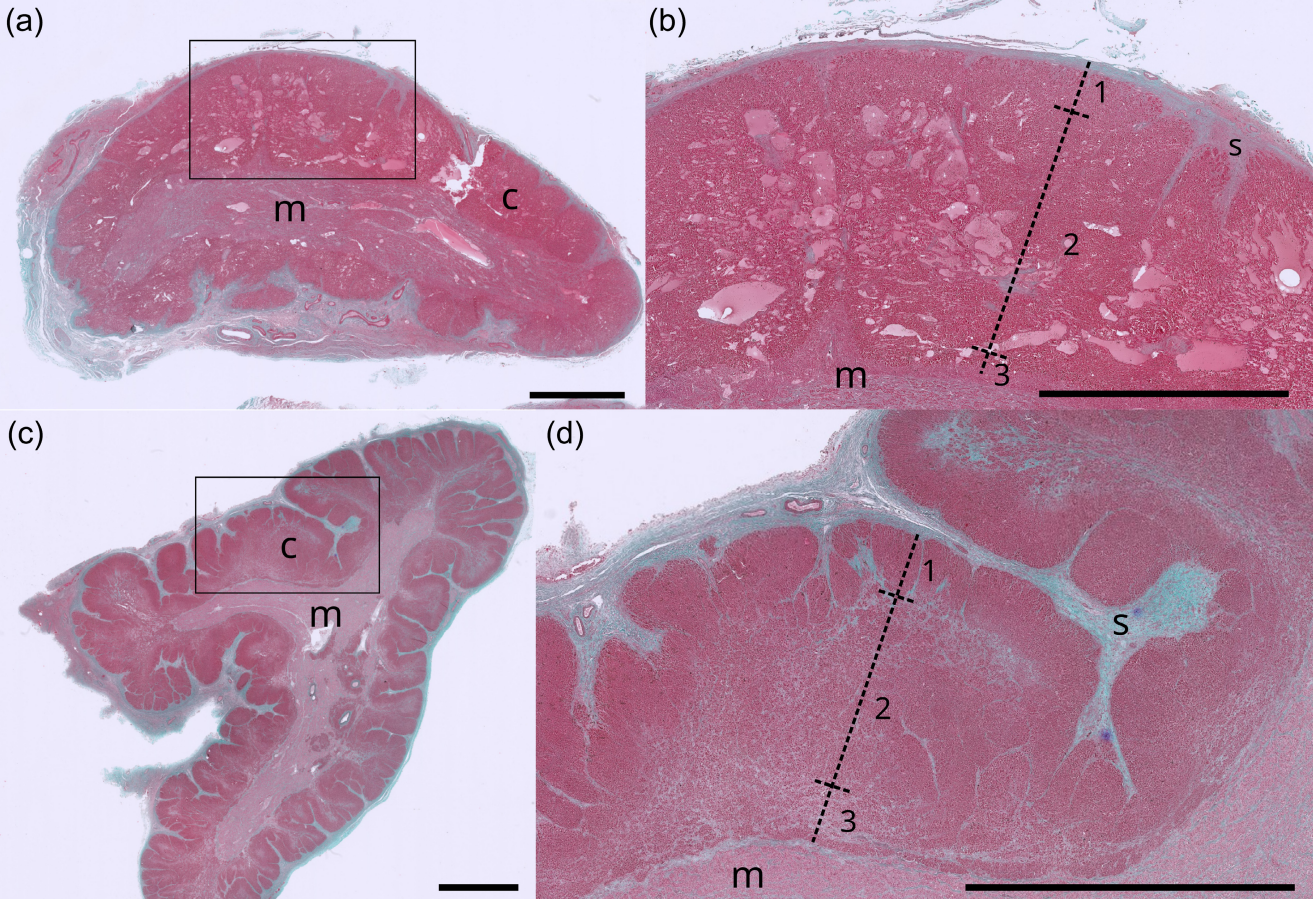
Microphotograph of the adrenal glands of (a, b) hourglass dolphin (*C. cruciger*) KS20‐20Lc and (c, d) spectacled porpoise (*P. dioptrica*) KS20‐07Pd. (a) and (c) display the whole gland, while (b) and (d) show a subdivision of the cortex into zona *glomerulosa* (1), *fasciculata* (2) and *reticularis* (3). C, cortex; M, medulla; S, septa. Scale bar = 500 μm. Masson's trichrome stain.

In both species, the adrenal glands were composed of a thick cortex (divided into *zona glomerulosa*, *fasciculata*, and *reticularis*) and a thin medulla. The adrenal glands of hourglass dolphins demonstrated a more ovoid shape compared to the spectacled porpoises, which were more pyramidal in shape. Microscopically, there were more septa in the spectacled porpoise compared to the hourglass dolphin, with the zona *fasciculata* comprising the thickest component (Figure 21). Details on the size and weight of the adrenal glands are reported in Table 12.

**TABLE 12:** Adrenal gland morphometry with measurements shown as length × width × diameter.

Parameter	Hourglass dolphin (*Cephalorhynchus cruciger*)		Spectacled porpoise (*Phocoena dioptrica*)		
	KS10‐28Lc	KS20‐20Lc	KS20‐07Pd	KS14‐37Pd/X2020.77	KS14‐45Pd/X2020.76
Adrenal R weight (g)	n/a	8	7	19.2	15
Adrenal R measurements (mm)	n/a	78 × 28 × 9	55 × 30 × 17	67 × 35 × n/a	n/a
Adrenal L weight (g)	n/a	7	8	18.4	11
Adrenal L measurements (mm)	n/a	52 × 23 × 7	51 × 34 × 10	55 × 26 × n/a	n/a

Abbreviation: L = left; R = right.

### 3.7 Urogenital system

#### 3.7.1 Kidneys

**FIGURE 22:**
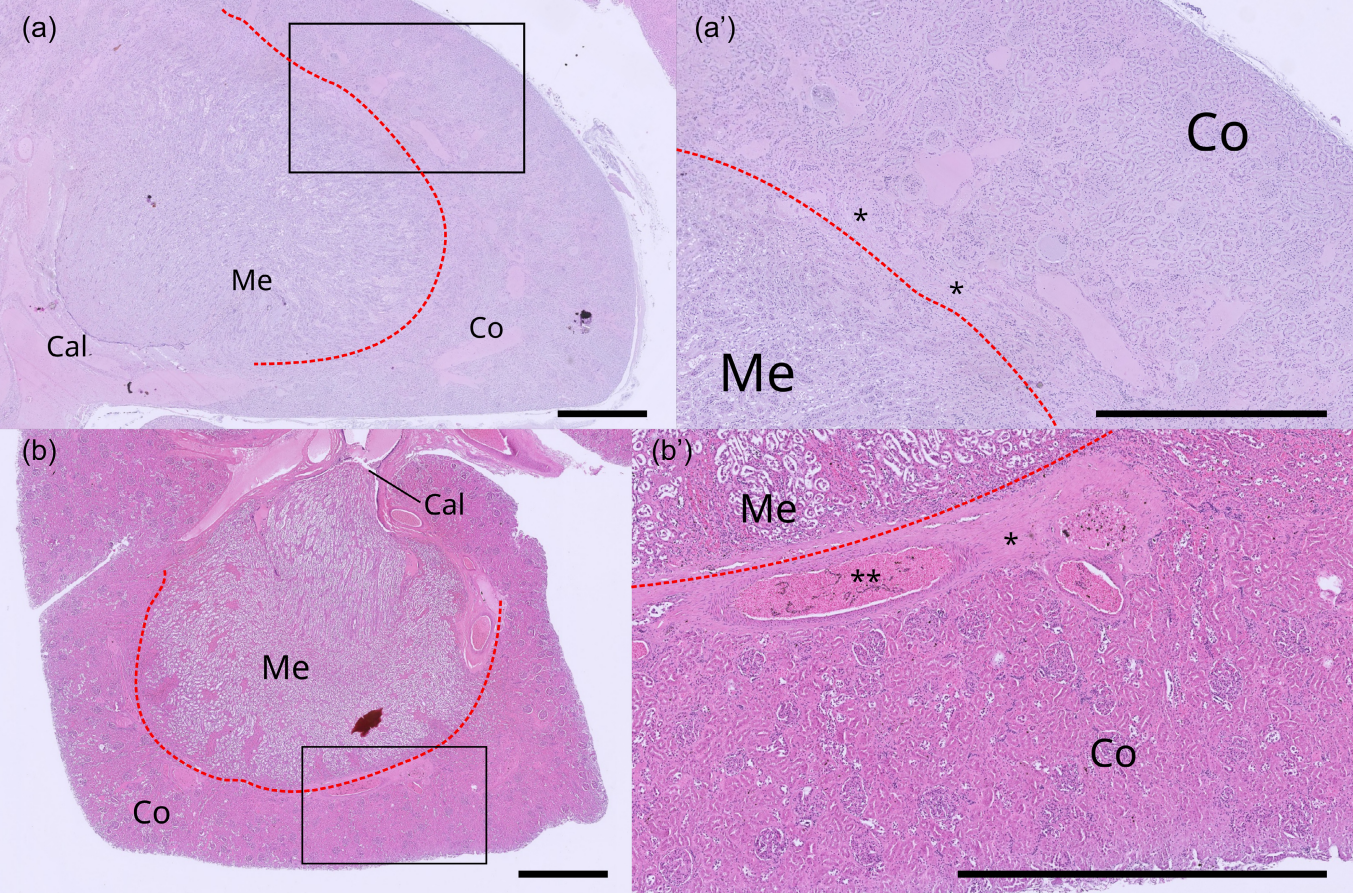
Microphotograph of reniculus of (a, a′) hourglass dolphin (*C. cruciger*) KS20‐20Lc and (b, b′) spectacled porpoise (*P. dioptrica*) KS20‐07Pd. (a) and (b) show the whole structure with the red dotted line dividing the cortex (Co) from the medulla (Me), which in turn collects the urine in the calyx (cal). (a′) and (b′) show the muscular basket (*) and renicular arterioles (**). Scale bar = 1 cm. Hematoxylin–eosin stain.

Each kidney in the hourglass dolphins had ca. 300 reniculi, similar to that of the bottlenose dolphin *Tursiops truncatus* (Cozzi et al., 2017). Renal structure was typical of cetaceans, with the muscular basket (*sporta perimedullaris*) and renicular arterioles dividing the cortex and medulla (Figure 22). Details on the morphometry of the kidneys are further summarized in Table 13.

**TABLE 13:** Morphometry of the kidneys. Measurements: Length × width × diameter.

	Hourglass dolphin (*Cephalorhynchus cruciger*)		Spectacled porpoise (*Phocoena dioptrica*)			
Parameter	KS10‐28Lc	KS20‐20Lc	KS20‐07Pd	KS14‐45Pd/X2020.76	KS14‐37Pd/X2020.77	KS15‐29Pd/VT3347
R K weight (g)	300	455	378	414	324	112.26
R K measurements (mm)	200 × 90 × 25	251 × 104 × 27	238 × 99 × 25	250 × 90 × 30	198 × 79 × 41.6	n/a
L K weight (g)	335	458	372	431	260.6	105.5
L K measurements (mm)	220 × 90 × 30	243 × 123 × 21	233 × 98 × 25	240 × 90 × 33	210 × 80 × 31.4	n/a
Bladder weight (g)	n/a	47	n/a	n/a	n/a	n/a
Bladder measurements (mm)	n/a	103 × 34 × 20	n/a	n/a	n/a	n/a

Abbreviations: K, kidney; L, left; R, right.

#### 3.7.2 Gonads

In KS15‐29Pd/VT3347, the left ovary weighed 1.9 g, was 15 mm in length, 10 mm in width, and 5 mm in diameter. The uterus was 30 mm in length and 15 mm in width, signaling an immature female.

**FIGURE 23:**
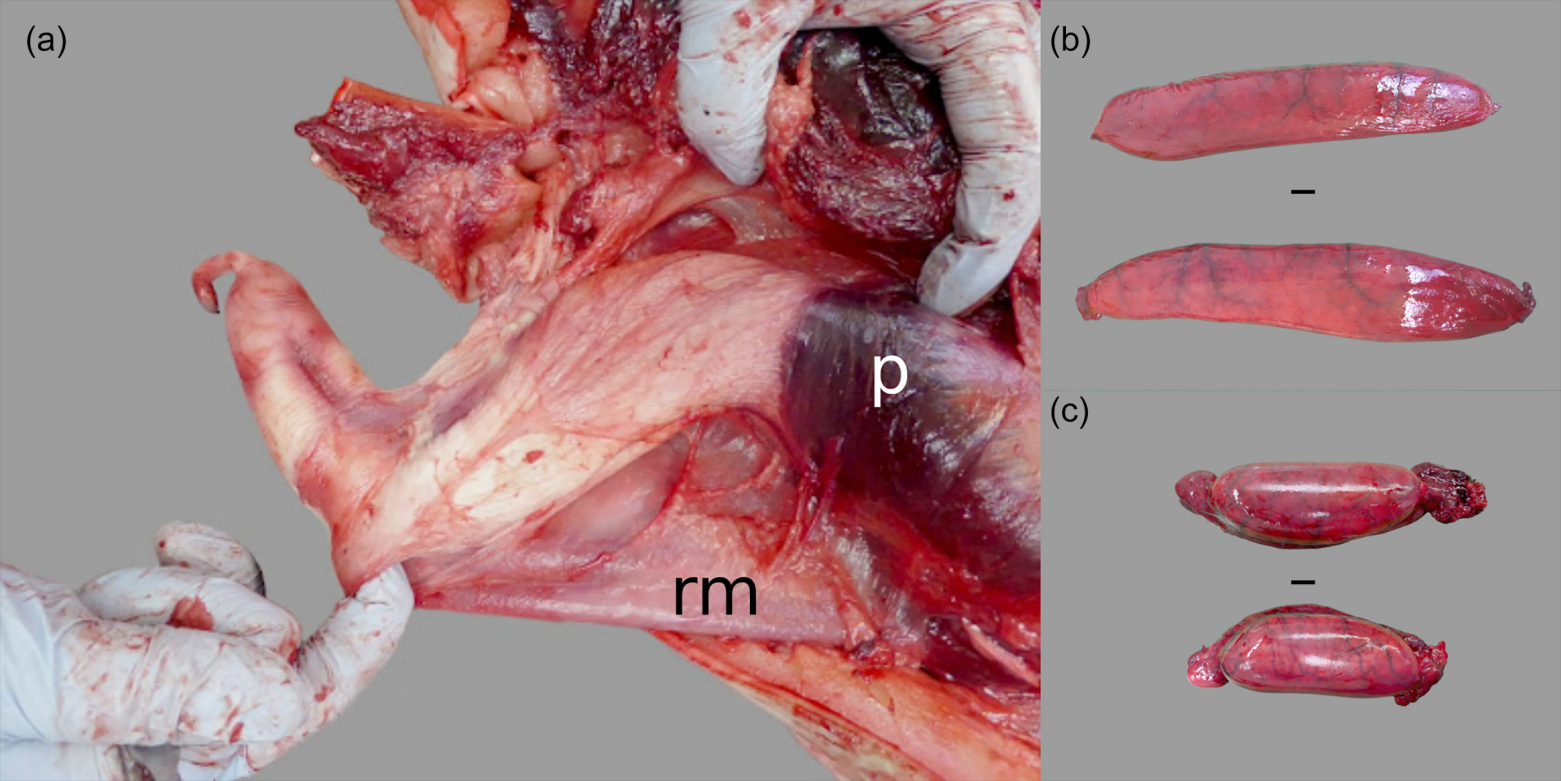
Male reproductive system: (a) Penis of hourglass dolphin (*C. cruciger*) KS20‐20Lc with retractor muscle (rm) and prostate (p) covered by the muscles ischiocavernosus and bulbospongiosus. The upper hand is holding soft tissue (partially resected muscle) surrounding the pelvic bone; (b) right (top) and left (bottom) testes with cranial pole on the left of hourglass dolphin KS20‐20Lc; (c) left (top) and right (bottom) testes with cranial pole on the left of spectacled porpoise KS20‐07Pd. Scale bar = 1 cm.

In males of both species, the penis was S‐shaped and displayed the retractor muscles. The apex was thin and the prostate gland clear (Figure 23).

In the five males, testes were large, as usual in adult cetaceans, with a white‐pearl color, and an epididymis running along the dorsal border of the testicle for most of its length. However, the testes were more elongated in the hourglass dolphins compared to the spectacled porpoise. The size of the testes in both species indicated that all males were sexually mature. In the case of KS20‐20Lc, sexual maturity was confirmed by the presence of spermatozoa on histological examination of the testis.

Details on the morphometry of the genital apparatus are reported in Table 14.

**TABLE 14:** Morphometry of the male genital apparatus.

Parameter	Hourglass dolphin (*Cephalorhynchus cruciger*)		Spectacled porpoise (*Phocoena dioptrica*)		
	KS10‐28Lc	KS20‐20Lc	KS20‐07Pd	KS14‐45Pd/X2020.76	KS14‐37Pd/X2020.77
R T + E weight (g)	315	434	323	n/a	119.9
R T − E weight (g)	267	369	276	n/a	n/a
R T measurements − length × width × depth (mm)	305 × 59	350 × 72 × 21	161 × 68 × 39	131 × 51 × n/a	150 × 50 × n/a
L T + E weight (g)	360	466	285	n/a	124.5
L T − E weight (g)	270	383	249	n/a	82.2
L T measurements − length × width × depth (mm)	285 × 55	375 × 74 × 19	152 × 61 × 39	138 × 50 × n/a	150 × 50 × n/a
Postanal hump measurements (cm)	4.3 × 28	6.5 × 5.2 × 28	n/a	n/a	n/a
Penis length (mm)	n/a	300	n/a	~190	n/a

*Note*: Measurements are reported as length × width × diameter (mm).

Abbreviations: E, epididymis; L, left; R, right; T, testis.

## 4 DISCUSSION

**FIGURE 24:**
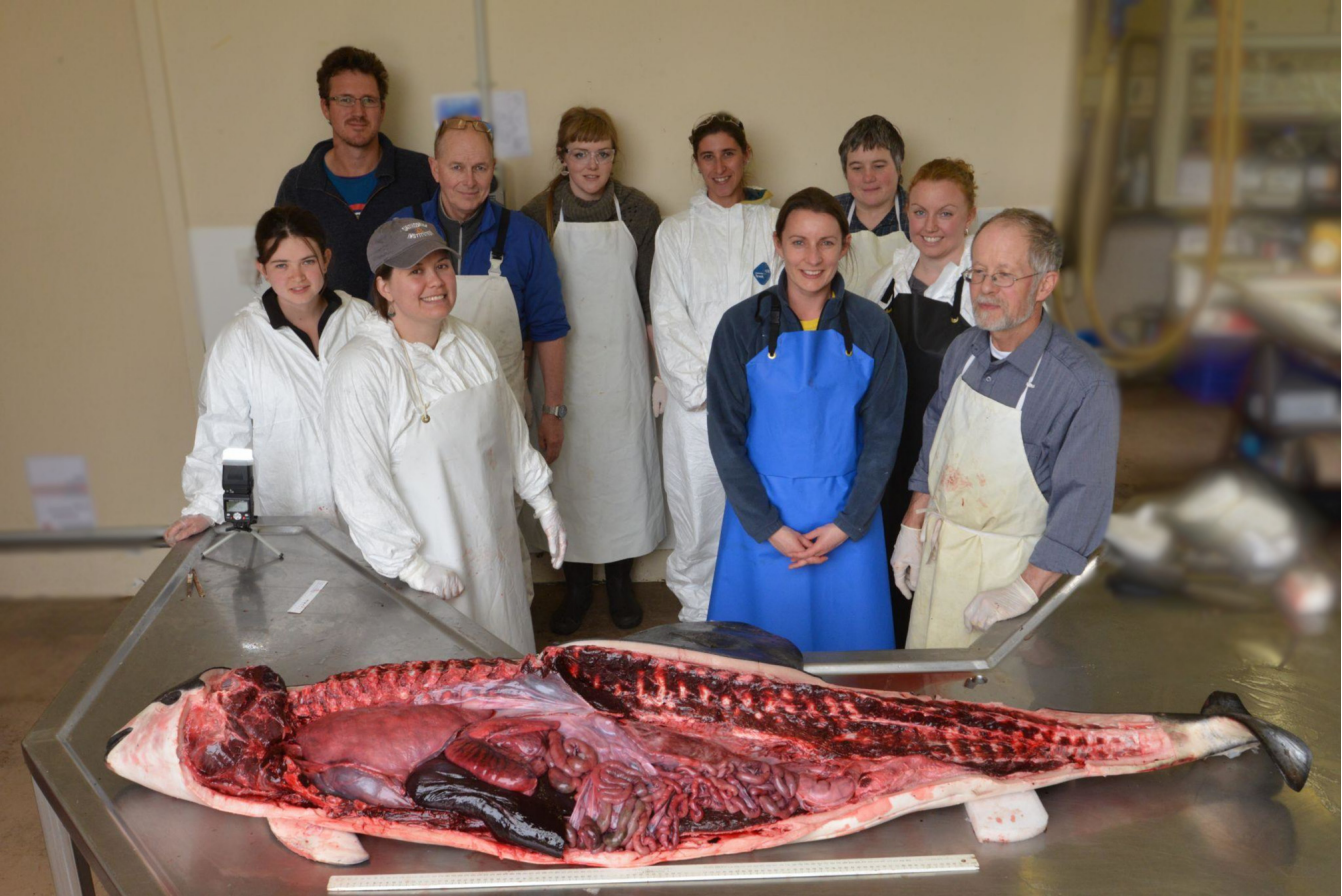
Dissection of spectacled porpoise KS14‐37Pd/X2020.77 with members from Massey University, the University of Otago, and Tūhura Otago Museum. Far Right—the Prof R. Ewan Fordyce FRSNZ (1953–2023) to whom this manuscript is posthumously dedicated.

The anatomical description of little known or endemic species is rarely for the sole purpose of describing a new species, but rather to offer insight on their biology, functional morphology, or evolutionary adaptations. Here, we provided an anatomical overview and description of two seldom reported species, the hourglass dolphin and the spectacled porpoise, using not only conventional photography and histology but also computed tomography including three‐dimensional reconstructions. This work was only possible thanks to the collaborative efforts of many individuals spanning multiple teams within and beyond Aotearoa, New Zealand (Figure 24).

The external appearance of both species reflected the description described in the literature: the hourglass dolphin is characterized by its unique white‐black pigmentation pattern on the flanks and the hooked dorsal fin; the spectacled porpoise is easy to recognize because of its large dorsal fin, the absence of a beak, and a white line surrounding the black eye spot. In terms of size, both species were within similar ranges of total body length as that described in the literature, although male hourglass dolphins weighed marginally less than what was previously reported for adult males (~90 kg). Skeletal features, including condylo‐basal length and dental characteristics, aligned with previously reported ranges for these species (Brownell & Donahue, 1999; Brownell Jr., 1999; Evans et al., 2001; Fernández et al., 2003; Gazitúa et al., 1999; Goodall et al., 1997; Pinedo et al., 2002). The anatomical features of other organs, such as the spleen, gastrointestinal tract, liver, adrenal glands, kidneys, and male reproductive systems, were similar to those of other odontocetes. This similarity reinforces the idea of a shared evolutionary lineage and common functional adaptations among cetaceans (Cozzi et al., 2017). This included the characteristic “keel” of the hourglass dolphin, which was composed of blubber and dense connective tissue. This feature, as in other delphinids such as Atlantic white‐sided dolphins (*Aethalodelphis acutus*; Fairley et al., 1990) or spinner dolphin (*S. longirostris*; Mead et al., 1980), might function similarly to that of a keel on a yacht, contributing to hydrodynamic stability by reducing roll and yaw during high‐speed swimming or, alternatively, it may be a sign of sexual dimorphism (Lammers, 2019).

While the pectoral fin of the hourglass dolphin was typical of other delphinids (Cozzi et al., 2017), the overall flipper shape in the spectacled porpoise appeared more rounded and tapered, resembling that of the sperm whale (*Physeter macrocephalus*). Specifically, the flipper length represented approximately 11% of the total body length in adults, which aligns with values reported for the sperm whale (~9.1%; Flower, 1868). However, the flipper skeletal anatomy did not fully align with that of the sperm whale, as the phalanges of the porpoise were more rounded in shape, rather than elongated as noted in sperm whales (Flower, 1868). These morphological characteristics may be associated with specific diving locomotor habits. A shorter, broader flipper may enhance maneuverability, allowing for fine‐scale changes in direction or rotation during swimming (Weber et al., 2014). However, given the absence of data on diving patterns of the spectacled porpoise, these observations need further morphofunctional investigation.

The respiratory anatomy revealed a lack of lobation and the presence of a right tracheal bronchus, consistent with other cetaceans (Cozzi et al., 2017; Fanning & Harrison, 1974). Although systematic lung measurements were not consistently taken and total lung capacity estimation was challenging due to the tools used (water displacement), qualitative evaluation indicated that both species appear to possess relatively large lungs for their body size. This observation would align with the “short dive, big lung” relationship observed by Piscitelli et al. (2010, 2013), suggesting that their lung size would be adapted for shallow diving behavior. However, our observations were notably based on gross dissection and imaging rather than systematic volumetric measurements, thus, no definitive conclusions can be drawn regarding the ecological function of lung size. Meanwhile, observation of the cardiocirculatory system suggests the topography and anatomy of the heart in both species aligns with that of other delphinids (Cozzi et al., 2017).

The dorsal fin of the spectacled porpoise presented unique characteristics, including its size and blood supply. The dorsal fin of other cetaceans studied in captivity has been demonstrated to be a heterogeneous thermoregulatory window, together with the fluke and flippers (Cozzi et al., 2017; Favilla et al., 2024; Meagher et al., 2002; Plön et al., 2018), allowing these animals to conserve or dissipate body heat as needed. In particular, vascularization of the dorsal fin is hypothesized to cool down the male gonads (Pabst et al., 1995; Plön et al., 2018; Rommel et al., 1992, 1993). Our observations revealed large vessels branching extensively throughout the dorsal fin in the spectacled porpoise, reaching the tip. These findings were consistent with those reported by Plön et al. (2018) in their studies of the Indo‐Pacific humpback dolphin (*Sousa plumbea*) and the Indo‐Pacific bottlenose dolphin (*Tursiops aduncus*). However, we noted a paucity of vessels in the cranial and caudal regions of the fin. This inhomogeneity may be due to technical limitations in detecting these vessels on CT or a true absence. Interestingly, it is worth noting that the Dall's porpoise (*Phocoenoides dalli*), a relative of the spectacled porpoise that also inhabits cold waters, possesses both a small dorsal fin and fluke, in addition to having a relatively thin blubber layer (Jefferson, 2018). Species that live in cold environments are unlikely to evolve wide and highly vascularized superficial structures that may dissipate heat (Ryding et al., 2021). This comparison suggests that further investigation is required to discern whether there are species‐specific differences in vascular system capacity of the dorsal fin to reduce heat loss and therefore, variability in thermoregulatory strategies among cetaceans. For example, could the large surface area of the spectacled porpoise dorsal fin allow blood flowing through its extensive vascular network to warm by maximizing exposure to sunlight? Further analysis involving multiple techniques, for example, MRI with contrast agents on the vasculature of the dorsal fin should be performed across sexes and age classes to enhance the quality of results. Alternatively, the large size of the dorsal fin of the spectacled porpoise could serve multiple non‐thermoregulatory functions. One possibility is that it plays a role in sexual display, since there is known sexual dimorphism (Jefferson et al., 2015), as also observed in other sexually dimorphic odontocetes such as pilot whales (*G. melas*; Betty et al., 2022). Another non‐thermoregulatory function could also be related to stability and control of lateral torsion during swimming, thus indirectly enhancing propulsive efficiency. Collectively, these characteristics highlight the many possible roles of the dorsal fin which may affect the ecology and survival of the spectacled porpoise.

To a lesser degree, the dorsal fin of the hourglass dolphin was also large with a wide surface area. However, the anatomical characteristics of vessels observed in the available CT images were unlike those of the spectacled porpoise. As for other cetaceans, its function is most likely related to thermoregulation. Its unique shape and size, present only in males, could also represent a sexual dimorphism trait (Jefferson et al., 2015). Alternatively, the combination of the dorsal fin with the shape and size of the caudal keel may also suggest it plays a role in stabilization during swimming.

Regardless of insights provided, our study faced several limitations regarding the opportunistic methodologies employed. For example, not all specimens were dissected in the same manner or imaged uniformly. Variation in CT scan quality arose from access to different machines and the advance of technology across the 10 years of specimen acquisition. More recent scans showed improved resolution compared to the earlier scans. The unattached large dorsal fin of spectacled porpoise KS14‐45Pd/X2020.76 hindered complete scans of the entire animal, making it difficult to count the lumbar vertebrae. Additionally, histological analyses were limited to only a selection of specimens, highlighting the need for greater consistency in future examinations of these rare and elusive species. Despite such challenges, the findings presented here support existing literature while documenting for the first time, the vascular pattern of the dorsal fin of the spectacled porpoise.

In conclusion, this anatomical study of the hourglass dolphin and spectacled porpoise demonstrated numerous similarities to other dolphins and porpoises as well as some peculiarities. Our findings underscore the need for further research into their adaptations to marine environments, reflecting evolutionary pressures that shaped their morphology and physiology. Further research is encouraged on their ecological roles and the potential impacts of environmental changes on their populations.

## AUTHOR CONTRIBUTIONS

**Jean‐Marie Graïc:** Conceptualization; investigation; writing – original draft; writing – review and editing; visualization; project administration; methodology. **Tommaso Gerussi:** Conceptualization; investigation; writing – original draft; writing – review and editing; visualization; project administration; methodology. **Bruno Cozzi:** Investigation; methodology; visualization; writing – review and editing; writing – original draft. **Rebecca M. Boys:** Conceptualization; investigation; writing – original draft; writing – review and editing; visualization; methodology; data curation. **Brian Chin Wing Kot:** Methodology; writing – review and editing. **Matthew R. Perrott:** Investigation; methodology; writing – review and editing. **Kane Fleury:** Data curation; writing – review and editing. **Tabris Yik To Chung:** Investigation; writing – review and editing. **Henry Chun Lok Tsui:** Investigation; writing – review and editing. **Emma Burns:** Writing – review and editing. **Trudi Webster:** Writing – review and editing; data curation; investigation. **Stuart Hunter:** Writing – review and editing; investigation; methodology. **Emma L. Betty:** Writing – review and editing; data curation; investigation. **Odette Howarth:** Data curation; writing – review and editing. **Carolina Loch:** Data curation; writing – review and editing. **Sophie White:** Data curation; writing – review and editing. **Steve Dawson:** Data curation; writing – review and editing. **William Rayment:** Data curation; writing – review and editing. **Ros Cole:** Writing – review and editing. **Derek Cox:** Writing – review and editing. **Tom Waterhouse:** Writing – review and editing. **Hannah Hendriks:** Writing – review and editing. **Anton van Helden:** Writing – review and editing. **Muriel Johnstone:** Conceptualization; writing – review and editing. **Ramari Oliphant Stewart:** Conceptualization; data curation; investigation; writing – review and editing. **R. Ewan Fordyce:** Data curation. **Karen A. Stockin:** Conceptualization; investigation; methodology; visualization; resources; supervision; writing – original draft; writing – review and editing; funding acquisition; project administration.

## FUNDING INFORMATION

KAS was supported by a Royal Society Te Apārangi Rutherford Discovery Fellowship. ELB was supported by the Colgan Foundation.

## CONFLICT OF INTEREST STATEMENT

The authors declare no potential conflict of interest.

## Data Availability

The data that support the findings of this study are available on request from the corresponding author. The data are not publicly available due to cultural and ethical restrictions.
